# Patterns of Use of Heated Tobacco Products: A Comprehensive Systematic Review

**DOI:** 10.2188/jea.JE20240189

**Published:** 2025-05-05

**Authors:** Marco Scala, Giulia Dallera, Giuseppe Gorini, Jérémie Achille, Anne Havermans, Clara Neto, Anna Odone, Luc Smits, Antonella Zambon, Alessandra Lugo, Silvano Gallus

**Affiliations:** 1 Department of Medical Epidemiology, Istituto di Ricerche Farmacologiche Mario Negri IRCCS, Milan, Italy; 2 Oncologic Network, Prevention and Research Institute (ISPRO), Florence, Italy; 3 French Agency for Food, Environmental and Occupational Health & Safety (ANSES), Maisons-Alfort, France; 4 Centre for Health Protection, National Institute for Public Health and the Environment (RIVM), Bilthoven, The Netherlands; 5 Department of Public Health, Experimental and Forensic Medicine, University of Pavia, Pavia, Italy; 6 Medical Direction, Fondazione IRCCS Policlinico San Matteo, Pavia, Italy; 7 Care and Public Health Research Institute, Department of Epidemiology, Maastricht University, Maastricht, The Netherlands; 8 Department of Statistics and Quantitative Methods, University of Milano-Bicocca, Milan, Italy; 9 Biostatistics Unit, IRCCS Istituto Auxologico Italiano, Milan, Italy

**Keywords:** heated tobacco products, heat-not-burn tobacco, dual use, public health, meta-analysis

## Abstract

**Introduction:**

Relative or absolute safety of heated tobacco products (HTPs) remains unknown, while independent literature suggests that these products do not favor tobacco control. We conducted a comprehensive systematic review and meta-analysis to evaluate HTP usage patterns and the effect of HTP use on conventional tobacco smoking (use transitions).

**Methods:**

We used Pubmed/MEDLINE, Embase, and the Cochrane Library to identify all articles published up to February 2022 on HTP use. For the present review, we included all representative cross-sectional studies dealing with HTP use, and all prospective cohort studies or cross-sectional studies on conventional tobacco smoking transitions due to HTP use. From 610 non-duplicate articles, 76 were eligible (71 cross-sectional and 5 prospective cohort studies).

**Results:**

Compared with young adults, HTP use was less frequent among middle-aged (15 studies; pooled odds ratio [OR] 0.59; 95% confidence interval [CI], 0.48–0.74) and older adults (12 studies; OR 0.17; 95% CI, 0.07–0.38). HTP use was more frequent among former (6 studies; OR 2.73; 95% CI, 1.03–7.25) and current smokers (12 studies; OR 14.53; 95% CI, 6.34–33.31). Overall, 68.3% of HTP users were dual users (*n* = 26). Eight studies (including 5 cohorts) showed that HTP users were more likely than non-users to start conventional cigarette smoking (2 studies; OR 6.31; 95% CI, 4.13–9.65), whereas current cigarette smokers using HTPs were less likely to quit (4 studies; OR 0.84; 95% CI, 0.80–0.89).

**Conclusion:**

We found that HTPs are specifically popular among young generations. More than two out of three HTP users are dual users. Prospective studies consistently show that, in real life, HTPs are not effective smoking-cessation tools.

## INTRODUCTION

Heated tobacco products (HTP) are tobacco sticks heated by an electric device to produce an aerosol containing nicotine.^1^^–^^3^ Philip Morris International (PMI) was the first transnational tobacco company to launch its HTP device, IQOS, in Italy and Japan in 2014.^4^ This device is currently available for sale in a large majority of high-income countries worldwide.^5^ Other best-selling HTP devices include Glo (by British American Tobacco [BAT]) and Ploom TECH (by Japan Tobacco International [JTI]), with both products being sold since 2016.

Although HTPs are promoted by the tobacco industry as a safer product compared to conventional tobacco, evaluation of their relative or absolute health effects is complex and thus far has been inconclusive. In fact, besides emitting lower concentrations of the same harmful substances produced by conventional cigarettes, HTPs release several other toxicological and potentially carcinogenic compounds that are not even emitted by conventional cigarettes.^6^ Moreover, no prospective data on the association between HTP use and disease incidence and mortality are available so far. Therefore, the only evidence-based conclusion regarding HTP safety is that these products are not safe for health.

Whereas e-cigarettes showed a certain success as smoking-cessation aids in clinical settings,^7^ no data on the effectiveness of HTPs are available from clinical trials.^8^ More importantly, in real life, HTPs can pose major public health problems, since these products, as well as e-cigarettes,^9^ appear to thwart—not favor—tobacco control.^10^ Despite these facts, in many countries HTPs enjoy large fiscal and regulatory benefits compared to conventional cigarettes,^11^ which substantially contributed to the spread of these products. A recent and comprehensive meta-analysis investigated the global prevalence of HTP use.^12^ From 45 studies conducted in 42 different countries, the authors identified growing estimates for lifetime (4.87%; 95% confidence interval [CI], 4.16–5.63%) and current use (1.53%; 95% CI, 1.22–1.87%), and non-negligible estimates also for daily HTP use (0.79%; 95% CI, 0.48–1.18%).

To our knowledge, there is no systematic review and meta-analysis in the literature examining patterns of HTP use. To fill this gap, we conducted a comprehensive systematic review of the literature in order to investigate patterns of HTP use and the effect of the use of these products on conventional tobacco smoking (use transitions).

## METHODS

### Systematic review

A systematic search of the scientific literature was conducted to retrieve all the available publications on HTPs (PROSPERO registration number: CRD42020137394). The search was performed on February 23, 2022 in Pubmed/MEDLINE, Embase and the Cochrane Library (the search strings are reported in eTable 1) and lead to the identification of 610 non-duplicate publications on HTPs. The objective of the present systematic review was to evaluate HTP use patterns and the effect of HTP use on conventional tobacco smoking. For this aim we considered as eligible articles: 1) primary publications providing data on use of HTP from representative cross-sectional studies, plus 2) primary publications evaluating the effect of HTP use on conventional tobacco smoking from cross-sectional studies and prospective studies. Two independent reviewers screened the 610 publications through a two-stage procedure (first title and abstract, second full-text) to identify all eligible articles. We excluded 336 non-eligible studies (eg, chemical/toxicological studies, in vivo or in vitro studies, or study protocols), 157 non-pertinent papers (eg, original studies or reviews focused on tobacco products other than HTPs), 11 papers showing prevalence estimates of HTP use in samples not representative of the general populations or of specific subgroups (eg, smokers or teenagers), 7 duplicate studies (ie, same data reported in more than one article), and 19 original articles focusing exclusively on perceptions and attitudes. After the inclusion of one additional article from six reviews on the issue and a further relevant article on the topic from other sources, we obtained 76 eligible publications (eFigure 1). The study followed the Preferred Reporting Items for Systematic Reviews and Meta-Analysis (PRISMA) reporting guidelines (eTable 2).

For each eligible study, we extracted the following relevant information using a standardized form in Microsoft Excel (Microsoft Corp, Redmond, WA, USA): first author, year of publication, country and year of conduction of the fieldwork, sample size, target population (ie, general population or specific subgroup), and results of the study including the prevalence of use (overall and stratified by socio-demographic characteristics), the proportion of dual users among current HTP users, and various risk measures (including odds ratios [OR], relative risk ratios [RRR], relative risks [RR], or prevalence ratios [PR], adjusted or crude with their corresponding 95% CIs).

### Meta-analyses

We conducted meta-analyses to provide pooled quantifications of specific aspects regarding the use of HTPs. In particular, we investigated the relationship between individual-level characteristics (ie, sex, age group, socio-economic status [SES], and use of conventional cigarette) and current use of HTPs. Moreover, the pooled estimate of the prevalence of dual users was analyzed. We used meta-analytic approaches only when considering articles on the general population, thus excluding studies limited to specific subgroups of the population, such as smokers or teenagers. Finally, we also projected pooled estimates of ORs of conventional cigarette smoking transitions as a consequence of HTP use.

#### Socio-demographic characteristics and smoking status

We included in this meta-analysis cross-sectional studies providing measures of association for the relationship between selected socio-demographic and smoking variables and the use of HTPs in the general population (ORs or their proxies) and corresponding 95% CIs, or raw data to compute the estimates and their confidence intervals. Adult study populations in various publications were re-classified into three categories: young adults (if the mean age of the group as reported in the original article was less than 40 years), middle-aged adults (mean age between 40 and 60 years), and older adults (mean age over 60 years). When necessary, we computed OR estimates to pool the ORs from various age categories using the Hamling method.^13^ We also applied the same methodology to pool categories of SES or conventional cigarette smoking, or to change reference categories.

#### Dual use

For the purpose of this meta-analysis, we included cross-sectional studies providing prevalence estimates of dual use among HTP users and corresponding 95% CIs, or raw data to compute their estimates and 95% CIs. Dual users were defined as subjects reporting being current smokers of conventional cigarettes and current HTP users. When the information on current HTP use was not reported, we defined dual users as subjects reporting to be current conventional cigarette smokers and ever HTP users. We included one publication considering any type of tobacco product users, not limited to conventional cigarette smokers only.^14^

#### Use transitions

We considered cross-sectional or prospective cohort studies providing risk estimates (OR, RR, or PR) for various outcomes related to conventional cigarettes smoking transitions (eg, smoking cessation, initiation, relapse, product switching) according to individual’s HTP use, and/or prevalence estimates of the proportion of HTP users that changed their habits of tobacco use. We provided pooled estimates of risk measures for starting smoking among never smokers, relapsing among former smokers, and quitting among current smokers, according to HTP use. Study quality was assessed using the Newcastle-Ottawa Scale (NOS) for cohort studies and the adapted version of the NOS for cross-sectional studies.^15^^,^^16^ NOS score for cohort studies ranges from 0 (poor quality) to 9 (good quality) and considers information on three broad categories: selection (maximum 4 points), comparability (maximum 2 points), and exposure (maximum 3 points). NOS score for cross-sectional studies ranges from 0 (poor quality) to 7 (good quality) and considers information on selection (3 points), comparability (2 points), and outcome (2 points). In this meta-analysis, high-quality studies were defined as those with NOS scores ≥7 for cohort studies and NOS score scores ≥6 for cross-sectional studies. No low-quality study was excluded from the meta-analysis.

#### Statistical analysis

We reported the prevalence of ever and current HTP use for adults and teenagers. If estimates from the same populations were reported in various articles, we considered only OR or prevalence estimate from the most recent paper or, if the year of publication was the same, from the article with the most complete data. We used random-effects meta-analytics models to obtain pooled results (OR and prevalence estimates). We used the I^2^ statistic to check for heterogeneity among studies.^17^ We performed stratified analysis by continent to check for geographic differences. For use transitions, we reported the main results provided by the original articles and summarized the evidence using a meta-analytic approach. We performed all statistical analyses using the software SAS (version 9.4; SAS Institute, Cary, NC, USAS) and R (version 4.3.0; R Foundation for Statistical Computing, Vienna, Austria).

## RESULTS

### Systematic review

The 76 eligible original publications on HTP use, including 71 cross-sectional studies and 5 cohort studies, were conducted between 2016 and 2022 in Asia (41 studies), Europe (22 studies), North America (15 studies), Oceania (3 studies), or Central America (2 studies). Characteristics of all these studies are reported in eTable 3.

We found 59 prevalence estimates of ever HTP users (ie, individuals who tried HTPs at least once in their lifetime) and 67 prevalence estimates of current HTP users (ie, individuals who used HTPs at least once during the last 30 days) in the adult population (eTable 4). Corresponding numbers for studies based on teenagers were 14 and 18, respectively (eTable 5).

**Figure 1.**
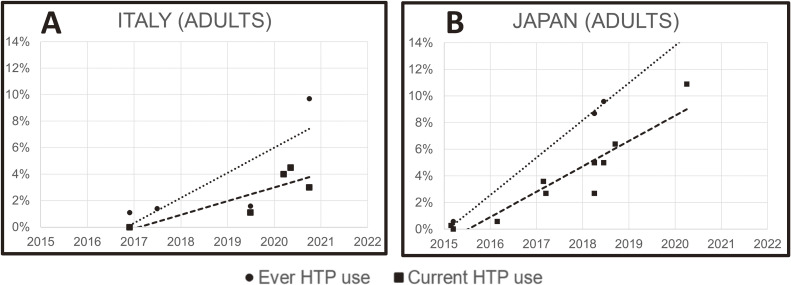
Trend of prevalence (%) of ever and current heated tobacco product use in the adult population in Italy (**A**) and Japan (**B**) from 2015 to 2021. HTP, heated tobacco product.

Among the 32 studies on adults, estimates of ever HTP use ranged from 0.1% in Japan (2015) to 23.9% in South Korea (2018) and estimates of current HTP use from 0.0% in Japan (2015) to 10.9% in Japan (2020). The only countries having three or more prevalence estimates in different years of current HTP use among adults were Japan (*n* = 10), Italy (*n* = 5), the United States (*n* = 3) and the United Kingdom (*n* = 5). The prevalence of HTP use increased substantially in the Japanese (annual percent change [APC] 2.1% between 2015 and 2020) and Italian general adult population (APC 1.0% between 2016 and 2020; Figure 1). In other countries, as in the USA (APC −0.1%) and the United Kingdom (APC 0.0%) no evident change in the use of HTPs was detected over time (between 2016 and 2020, data not shown in figures). For several other countries, the prevalence estimates of HTP use were scarce and inconsistent, thus not allowing us to define a country-specific trend of HTP use through the years, particularly for young people. Among the 17 studies on teenagers, estimates of ever HTP use ranged from 0.7% in the United States (2019–2020) to 11.3% in Guatemala (2019) and estimates of current HTP use from 0.2% in the United States (2019–2020) to 2.9% in Guatemala (2019).

### Meta-analyses

#### Socio-demographic characteristics and smoking status

**Figure 2.**
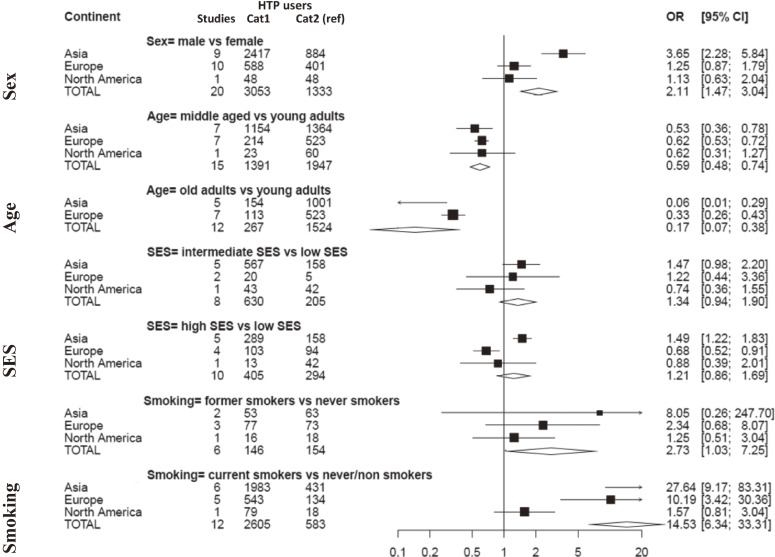
Forest plot of pooled odds ratios for current heated tobacco product use among adults according to different socio-demographic exposures (sex, age, socio-economic status, and smoking status), overall and stratified by continent Cat1, first category of the variable of interest (e.g., male for sex); Cat2, second and reference category of the variable of interest (e.g., female for sex) CI, confidence interval; HTP, heated tobacco product; OR, odds ratio; SES, socio-economic status.

Twenty-two studies were included in the meta-analysis on the relationship between demographic and socio-economic factors and HTP use (Figure 2 and eFigures 2, eFigure 3, eFigure 4, eFigure 5, eFigure 6, eFigure 7 and eFigure 8). We found that current HTP use was significantly related to being male (20 studies; OR for male vs female 2.11; 95% CI, 1.47–3.04), a young adult (15 studies; OR for middle-aged vs young adults 0.59; 95% CI, 0.48–0.74 and 12 studies; OR for older vs young adults 0.17; 95% CI, 0.07–0.38), and a former or current conventional cigarette smoker (6 studies; OR for former vs never smokers 2.73; 95% CI, 1.03–7.25 and 12 studies; OR for current vs never/non-smokers 14.53; 95% CI, 6.34–33.31). Globally, HTP use was not significantly associated with SES.

#### Dual use

**Figure 3.**
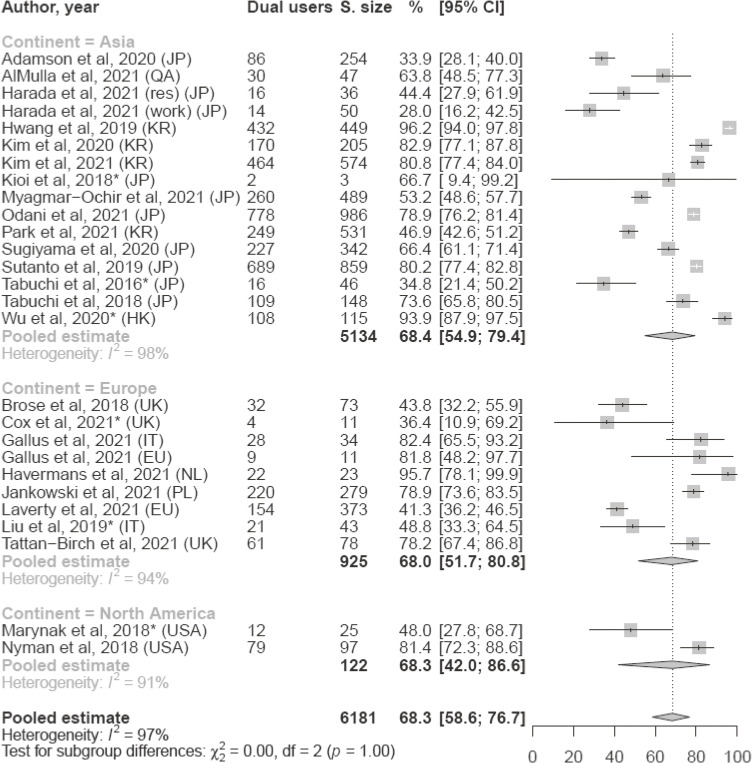
Forest plot of study-specific and pooled prevalence (%) of dual use of heated tobacco products (HTP) and conventional cigarettes among adult HTP users, overall and stratified by continent ^*^in the study dual users were defined as current conventional cigarette smokers and ever HTP users; CI, confidence interval; S. Size, Sample Size; EU, Europe (different countries); HK, Hong Kong; IT, Italy; JP, Japan; KR, South Korea; NL, Netherlands; PL, Poland; QA, Qatar; res, resident; UK, United Kingdom; USA, United States of America; work, work population.

Twenty-six studies were included in the meta-analysis on the prevalence of dual users among HTP users. We found that globally 68.3% of HTP users were dual users (95% CI, 58.6–76.7%, Figure 3), with prevalence estimates among all included studies ranging between 28.0% (95% CI, 16.2–42.5%) and 96.2% (95% CI, 94.0–97.8%). The prevalence was consistent through continents, with a 68.4% prevalence of dual users in Asia (95% CI, 54.9–79.4%; 16 estimates from 15 studies), 68.3% in North America (95% CI, 42.0–86.62%; studies), and 68.0% in Europe (95% CI, 51.7–80.89%; studies).

#### Use transitions

We found eight articles showing data on conventional cigarettes smoking transitions according to HTP use (Table 1). Two prospective studies and one cross-sectional study consistently showed that among never-smokers, HTP users were more prone to start conventional cigarette smoking than non-users. The pooled OR among never smokers was 6.31 (95% CI, 4.13–9.65; 2 studies; eFigure 9). Among former cigarette smokers, three prospective studies and one cross-sectional reported estimates on the association between current HTP use and relapsing in smoking conventional cigarettes. The pooled OR of relapsing among former smokers for current HTP users vs non/never-users was 2.15 (95% CI, 0.89–5.21; 3 studies; eFigure 10). A potential favorable effect of HTPs as a smoking cessation tool was refuted in all the seven studies conducted on conventional tobacco smokers (four cohort studies and three cross-sectional). The pooled OR of quitting smoking among current smokers for current HTP users vs non/never-users was 0.84 (95% CI, 0.80–0.89; 4 studies; eFigure 11). Quality assessment of included cohort and cross-sectional studies is shown in eTable 6 and eTable 7, respectively. None of the cohort studies (0/5) or the cross-sectional studies (0/21) achieved a high-quality score on the Newcastle-Ottawa scale.

**Table 1.** Main results from eight original publications providing data on heated tobacco products (HTP) and conventional cigarettes use transitions included in the systematic review

First author, year (reference)	Country	Year of data collection	Sample size	Population	Type of study	Type of endpoint	Endpoint	Main results
Gallus et al, 2022^10^	Italy	2020 (∼7 months of follow-up)	3,185	Adults	Prospective cohort study	Initiation	Among never smokers, RR of starting smoking conventional cigarettes for current vs never HTP users	RR of starting smoking = 5.80 (95% CI, 3.65–9.20)
						Relapse	Among former smokers, RR of relapsing for current vs never HTP users;	RR of relapsing = 3.32 (95% CI, 2.05–5.37)
						Cessation	Among current smokers, RR of continuing smoking for current vs never HTP users	RR of continuing smoking = 1.17 (95% CI, 1.10–1.23)
Kanai et al, 2021^29^	Japan	2018 (∼10 months of follow up)	158	Tobacco users at a manufacturing company	Prospective cohort study	Cessation	Among current smokers, RR of quitting smoking for current HTP users vs HTP non-users	RR of quitting smoking = 0.77 (95% CI, 0.61–0.97)
Luk et al, 2021^30^	Hong Kong	2018 (∼6 months of follow up)	1,213	Daily smokers	Prospective cohort study	Cessation	Among current smokers, PR of smoking abstinence at 3 months for current HTP users vs HTP non-users	PR of smoking abstinence at 3 months = 0.81 (95% CI, 0.43–1.52)
						Cessation	Among current smokers, PR of smoking abstinence at 6 months for current HTP users vs HTP non-users	PR of smoking abstinence at 6 months = 1.08 (95% CI, 0.63–1.85)
Matsuyama et al, 2022^31^	Japan	2019 (∼12 months of follow up)	5,947	Former or never combustible cigarette smokers	Prospective cohort study	Relapse	Among former smokers who recently quit, OR of relapsing for current HTP users vs HTP non-users	OR of relapsing = 0.59 (95% CI, 0.28–1.26)
						Relapse	Among former smokers who quit for one year or more, OR of relapsing for current HTP users vs HTP non-users	OR of relapsing = 2.80 (95% CI, 1.42–5.52)
						Initiation	Among never smokers, OR of starting smoking conventional cigarettes for current HTP users vs HTP non-users	OR of starting smoking = 9.95 (95% CI, 3.39–29.2)
Xia et al, 2022^32^	Hong Kong	2016–2019 (∼6 months of follow up)	579	Young Chinese smokers who want to quit	Prospective cohort study	Cessation	Among current smokers, RR of smoking abstinence at 1 week for current HTP users vs HTP non-users	RR of smoking abstinence at 1 week = 1.01 (95% CI, 0.79–1.28)
						Cessation	Among current smokers, RR of smoking abstinence at 1 month for current HTP users vs HTP non-users	RR of smoking abstinence at 1 month = 0.84 (95% CI, 0.66–1.07)
						Cessation	Among current smokers, RR of smoking abstinence at 3 months for current HTP users vs HTP non-users	RR of smoking abstinence at 3 months = 0.50 (95% CI, 0.29–0.86)
						Cessation	Among current smokers, RR of smoking abstinence at 6 months for current HTP users vs HTP non-users	RR of smoking abstinence at 6 months = 0.47 (95% CI, 0.24–0.91)
						Relapse	Among former smokers, RR of relapsing during the follow-up for current HTP users vs HTP non-users	RR of relapsing = 4.56 (95%-CI; 1.17–17.8)
Adamson et al, 2020^33^	Japan	2018	4,154	Population aged 20+ living in Sendai, Tokyo and Osaka	Representative cross-sectional study	Cessation	Proportion of HTP dual users according to the change in the status of smoking conventional cigarettes or HTPs	79.2% of dual users in the previous 12 months are current conventional cigarette smokers; 4.1% are former tobacco users
Gallus et al, 2021^34^	Italy	2019	3,120	Population aged 15+	Representative cross-sectional study	Initiation/relapse, cessation	Proportion of HTP ever users according to the change in the status of smoking conventional cigarettes	19.1% of ever HTP users started or re-started smoking conventional cigarette; 14.6% quit smoking; 66.3% did not change their smoking habit
Koyama et al, 2021^35^	Japan	2020	5,120	Tobacco users	Non-Representative cross-sectional study	Cessation	Among ever conventional cigarette smokers, PR of quitting smoking any type of tobacco for smokers who switched from conventional cigarettes to HTPs vs smokers who did not switch	PR of quitting smoking any type of tobacco = 0.15 (95% CI, 0.04–0.58)

CI, confidence interval; HTP, heated tobacco product; OR, odds ratio; PR, prevalence ratio; RR, relative risk.

## DISCUSSION

Our comprehensive systematic review and meta-analysis shows that HTP use among adults has increased substantially in Japan and Italy between 2016 and 2020, while in other European or American countries current use has not significantly changed. HTP users are more likely to be men, young adults, and current or former smokers. Moreover, two thirds of HTP users are dual users consuming both HTPs and conventional cigarettes. Current HTP use among the teenager population is also not negligible. Evidence from eight studies (including five prospective cohort studies) dealing with use transitions does not support these products as effective smoking cessation tools. In contrast, HTP users are more likely to start conventional cigarette smoking and less likely to quit conventional cigarettes.

In agreement with a previous systematic review on the issue, we observed a growing trend in HTP use in some countries, but not all.^12^ We observed impressive increase trends in Japan and Italy between 2016 and 2020. Notably, these countries have an extremely high level of tobacco industry interference index^18^ and were those chosen as pilot countries to launch the first HTPs for the Asian and Western markets, respectively.

Overall, we found that HTPs were more frequently used by men than women. However, this pattern was mainly present in studies from Asian countries. No difference in HTP use between men and women was observed in some countries in a more advanced stage of the tobacco epidemic,^19^ including the United States and several European countries. This finding is in agreement with a systematic review on HTP use, showing that the prevalence of current use in men (5.8%; 95% CI, 3.6–8.5%) was higher than in women (1.6%; 95% CI, 1.0–2.4%) in the Western Pacific Region, but not in the European Region or the Americas Region.^12^ This gender gap in HTP use mirrors the well-known gap for conventional tobacco products, although more pronounced for conventional cigarettes, particularly in Western Pacific countries, where only one in 18 tobacco users is female.^20^

The most frequent consumers of HTPs are young adults. Our results are consistent with another systematic review in which the percentage of ever users was higher in the younger population (5.3%; 95% CI, 4.4–6.2% for adolescents and 2.5%; 95% CI, 0.8–5.0% for adults).^12^ The prevalence of current use of these products among teenagers is not negligible in many countries, including Guatemala (2.9% in 2019), South Korea (2.6% in 2019), Taiwan (2.3% in 2018), and Italy (2.0% in 2018). Indeed, both the packaging and the technological components make these devices particularly attractive for teenagers,^21^^,^^22^ and the wide variety of flavors is appealing to young non-smokers, similar to electronic cigarettes, as the World Health Organization warns.^23^ Moreover, young people are the target population of the advertisements of these products, and kids and adolescents come into contact with uncontrolled HTPs advertisement on social media platforms.^24^^,^^25^

Socio-economic status does not emerge as a relevant influencing factor for HTP use in the global analysis, but we observed some differences between continents. We hypothesized that the high initial cost of these devices might discourage potential consumers with lower economic means. Indeed, some studies from the literature suggest that higher socio-economic classes were associated with the highest percentage of current HTP use.^26^^–^^28^ From our meta-analysis, we show that this is valid in Asia but not in Europe, where people with higher SES are less likely to use HTPs than people with lower SES.

If HTPs were an effective smoking cessation tool, we would expect the large majority of HTP users to be ex-smokers who successfully quitted due to a complete switch from conventional cigarette to HTP. On the contrary, current smokers are by far the slice of population most frequently using HTPs. Accordingly, we found that two out of three HTP users are dual users. This is of great concern because among current smokers, HTPs likely represent a compensative measure for smoking in settings where conventional cigarettes are banned, thus increasing the occasions for daily nicotine intake. These products are less frequently used by ex-smokers. Since our evidence comes from cross-sectional studies, it is difficult to infer if HTP users among former smokers had profited or not from their HTP use to quit smoking. However, a study conducted in 11 European countries showed that half of the former smokers using HTPs had quit smoking before HTPs were launched in their local markets.^26^ This indicates that a large proportion of former smokers using HTPs are not smokers who switched to HTPs to reduce their harm, but people who had already quit smoking and started using a tobacco product again with the entrance in commerce of HTPs. In order to assess how the prevalence of dual use changes in relation to the increase in the use of HTPs, future studies should evaluate the temporal trends of dual use in specific countries with a high prevalence of HTP use.

Eight studies^10^^,^^29^^–^^35^ (three cross-sectional and five cohort studies) provided data on cigarette smoking transitions according to HTP use, thus showing the change in the use of conventional cigarette over time based on individual-level use of HTPs. Despite the high heterogeneity in the study measures (risk measures or prevalence estimates) and the study populations (smokers or non-smokers) among the few studies available on the issue, we were able to provide pooled estimates quantifying the effect of HTP use on conventional cigarette smoking. All the publications consistently showed that dual users of HTPs and conventional cigarettes were less prone to quit conventional tobacco than exclusive smokers. Furthermore, HTP users were more inclined to start conventional cigarette consumption than HTP non-users, and three prospective studies showed that among former smokers, HTP users were more likely to relapse, with the possible exception of recent quitters, for whom no statistically significant association was found. Specific data on changes in conventional cigarette smoking intensity among HTP users were not systematically reported in the included studies, and none of the prospective cohort studies focused on this issue. This factor should be analyzed in future studies on HTPs. The available evidence therefore confirms that HTPs represent a gateway to smoking initiation and rejects the hypothesis of HTPs as effective tools to quit conventional cigarette smoking.

To our knowledge, this is the most comprehensive systematic review and meta-analysis on the patterns and transitions of HTP use globally. Our search strategy might have some limitations, but we minimized them by building a broad search string, including various terms referring to HTPs and performing the search on various scientific databases. We considered only scientific articles published in peer-reviewed journals, excluding information available in the grey literature and in other official reports.

Some other limitations are inherent to the characteristics of the original studies included in our systematic review and meta-analysis. First, most of the studies are cross-sectional studies, so we could not infer the causal associations between specific factors on HTP use. Furthermore, the definition of HTP use was inconsistent among studies, likely contributing, together with the differences between study populations and socio-demographic characteristics influencing HTP use, to the high heterogeneity observed in each meta-analysis. The quality of the cross-sectional studies included in the meta-analysis on the association between HTP use and socio-demographic characteristics, and of the cohort studies included in the meta-analysis on use transitions was assessed using the NOS quality scale (and the adapted version for cross-sectional studies), which is the most widely recognized tool for assessing the quality of observational studies.^15^^,^^16^ None of the studies achieved a high-quality score. In fact, the NOS scale captures the quality of the report rather than the quality of the study, while the scoring of most of its items is somewhat subjective and can vary depending on the type of follow-up, exposure, and outcome measured. As a result, in some cases it is difficult to obtain high scores even when the studies are of good quality.

In conclusion, our systematic review shows robust evidence of frequent use of HTPs among current smokers, who continue smoking conventional cigarettes, and among younger individuals and adolescents. The widespread use of HTPs among young people contradicts tobacco companies’ claims that these devices are designed only for heavy smokers, and confirms that the growing spread of HTP use represents a severe public health issue. More independent studies need to be conducted to obtain updated information to better understand the global trends and patterns of HTP use. The current evidence is strong enough to recommend to repeal the fiscal and regulatory benefits HTPs have compared to conventional cigarettes, to protect young adults and adolescents.

## SUPPLEMENTARY MATERIAL

eTable 1. Search strings used in various sources for the conduction of the systematic review

eTable 2. PRISMA 2020 checklist

eTable 3. Characteristics of 76 original articles providing data on the use of heated tobacco products (HTP) included in the review, with corresponding information contributing to the systematic review

eTable 4. Prevalence of use of heated tobacco products (HTP) among adults by country

eTable 5. Prevalence of use of heated tobacco products (HTP) among teenagers by country

eTable 6. Quality evaluation of the 5 cohort studies included in the meta-analysis on use transitions using the New-Castle Ottawa (NOS) scale

eTable 7. Quality evaluation of the 21 cross-sectional studies providing results on the association between heated tobacco products use and socio-demographic characteristics (sex, age, socio-economic status, use of conventional cigarette) using the New-Castle Ottawa scale adapted for cross-sectional studies

eFigure 1. Flowchart of the systematic review

eFigure 2. Forest plot of pooled odds ratio for current heated tobacco product use among adults according to sex (male vs female), overall and stratified by continent

eFigure 3. Forest plot of pooled odds ratios for current heated tobacco product use among adults according to age group (middle aged vs young adults), overall and stratified by continent

eFigure 4. Forest plot of pooled odds ratios for current heated tobacco product use among adults according to age group (old adults vs young adults), overall and stratified by continent

eFigure 5. Forest plot of pooled odds ratios for current heated tobacco product use among adults according to socio-economic status (intermediate vs low), overall and stratified by continent

eFigure 6. Forest plot of pooled odds ratios for current heated tobacco product use among adults according to socio-economic status (high vs low), overall and stratified by continent

eFigure 7. Forest plot of pooled odds ratios for current heated tobacco product use among adults according to cigarette smoking status (former vs never smokers), overall and stratified by continent

eFigure 8. Forest plot of pooled odds ratios for current heated tobacco product use among adults according to cigarette smoking status (current vs non/never smokers), overall and stratified by continent

eFigure 9. Forest plot of pooled odds ratios for conventional cigarette smoking initiation among never smokers according to heated tobacco product use (current vs non/never users)

eFigure 10. Forest plot of pooled odds ratios for conventional cigarette smoking relapse among former smokers according to heated tobacco product use (current vs non/never users)

eFigure 11. Forest plot of pooled odds ratios for conventional cigarette smoking cessation among current smokers according to heated tobacco product use (current vs non/never users)
