# Supplementary material for: Patterns of Use of Heated Tobacco Products: A Comprehensive Systematic Review
Source: J Epidemiol. 2025 May 5;35(5):213–21. doi: 10.2188/jea.JE20240189 (PMC11979348; doi:10.2188/jea.JE20240189)
Supplement: Supplementary file 1 [file je-35-213-s001.pdf]

eTable 1. Search strings used in various sources for the conduction of the systematic review

Source	Date	String used	Filters	N ref
**Pubmed**	23/2/2022	"heated tobacco" OR "heat-not-burn" OR IQOS OR ploom[tiab] OR "heated cigarette" OR "tobacco heating"	English	589
**Embase**	23/2/2022	('heated tobacco':ab,ti OR 'heat-not-burn':ab,ti OR iqos:ab,ti OR ploom:ab,ti OR 'heated cigarette':ab,ti OR 'tobacco heating':ab,ti) NOT [medline]/lim AND [english]/lim AND (article:it OR review:it)	Not Pubmed English Article or review	71
**Cochrane Library**	23/2/2022	"heated tobacco" OR "heat-not-burn" OR IQOS OR ploom OR "heated cigarette" OR "tobacco heating"	Title Abstract Keywords	2
		Duplicates		-52
**Total**	23/2/2022			610

eTable 2. PRISMA 2020 checklist

Section and Topic	Item #	Checklist item	Location where item is reported
**TITLE**			
Title	1	Identify the report as a systematic review.	1
**ABSTRACT**			
Abstract	2	See the PRISMA 2020 for Abstracts checklist.	2
**INTRODUCTION**			
Rationale	3	Describe the rationale for the review in the context of existing knowledge.	3
Objectives	4	Provide an explicit statement of the objective(s) or question(s) the review addresses.	3
**METHODS**			
Eligibility criteria	5	Specify the inclusion and exclusion criteria for the review and how studies were grouped for the syntheses.	4
Information sources	6	Specify all databases, registers, websites, organisations, reference lists and other sources searched or consulted to identify studies. Specify the date when each source was last searched or consulted.	3-4, eTable 1
Search strategy	7	Present the full search strategies for all databases, registers and websites, including any filters and limits used.	3-4, eTable 1
Selection process	8	Specify the methods used to decide whether a study met the inclusion criteria of the review, including how many reviewers screened each record and each report retrieved, whether they worked independently, and if applicable, details of automation tools used in the process.	4
Data collection process	9	Specify the methods used to collect data from reports, including how many reviewers collected data from each report, whether they worked independently, any processes for obtaining or confirming data from study investigators, and if applicable, details of automation tools used in the process.	4
Data items	10a	List and define all outcomes for which data were sought. Specify whether all results that were compatible with each outcome domain in each study were sought (e.g. for all measures, time points, analyses), and if not, the methods used to decide which results to collect.	4
	10b	List and define all other variables for which data were sought (e.g. participant and intervention characteristics, funding sources). Describe any assumptions made about any missing or unclear information.	4-5
Study risk of bias assessment	11	Specify the methods used to assess risk of bias in the included studies, including details of the tool(s) used, how many reviewers assessed each study and whether they worked independently, and if applicable, details of automation tools used in the process.	NA
Effect measures	12	Specify for each outcome the effect measure(s) (e.g. risk ratio, mean difference) used in the synthesis or presentation of results.	4-5
Synthesis methods	13a	Describe the processes used to decide which studies were eligible for each synthesis (e.g. tabulating the study intervention characteristics and comparing against the planned groups for each synthesis (item #5)).	4-5
	13b	Describe any methods required to prepare the data for presentation or synthesis, such as handling of missing summary statistics, or data conversions.	5
	13c	Describe any methods used to tabulate or visually display results of individual studies and syntheses.	4-5
	13d	Describe any methods used to synthesize results and provide a rationale for the choice(s). If meta-analysis was performed, describe the model(s), method(s) to identify the presence and extent of statistical heterogeneity, and software package(s) used.	4-5-6

Section and Topic	Item #	Checklist item	Location where item is reported
	13e	Describe any methods used to explore possible causes of heterogeneity among study results (e.g. subgroup analysis, meta-regression).	5
	13f	Describe any sensitivity analyses conducted to assess robustness of the synthesized results.	NA
Reporting bias assessment	14	Describe any methods used to assess risk of bias due to missing results in a synthesis (arising from reporting biases).	NA
Certainty assessment	15	Describe any methods used to assess certainty (or confidence) in the body of evidence for an outcome.	NA
**RESULTS**			
Study selection	16a	Describe the results of the search and selection process, from the number of records identified in the search to the number of studies included in the review, ideally using a flow diagram.	4,6, eFigure 1
	16b	Cite studies that might appear to meet the inclusion criteria, but which were excluded, and explain why they were excluded.	4, eFigure 1
Study characteristics	17	Cite each included study and present its characteristics.	eTable 3
Risk of bias in studies	18	Present assessments of risk of bias for each included study.	NA
Results of individual studies	19	For all outcomes, present, for each study: (a) summary statistics for each group (where appropriate) and (b) an effect estimate and its precision (e.g. confidence/credible interval), ideally using structured tables or plots.	Table 1, eTables 4 and eTable 5
Results of syntheses	20a	For each synthesis, briefly summarise the characteristics and risk of bias among contributing studies.	NA
	20b	Present results of all statistical syntheses conducted. If meta-analysis was done, present for each the summary estimate and its precision (e.g. confidence/credible interval) and measures of statistical heterogeneity. If comparing groups, describe the direction of the effect.	6-7, Figures 1-2-3, Table 1, eFigure 2, eFigure 3, eFigure 4, eFigure 5, eFigure 6, eFigure 7, eFigure 8, eFigure 9, eFigure 10, and eFigure 11
	20c	Present results of all investigations of possible causes of heterogeneity among study results.	Figure 3, eFigure 2, eFigure 3, eFigure 4, eFigure 5, eFigure 6,

Section and Topic	Item #	Checklist item	Location where item is reported
			eFigure 7, and eFigure 8
	20d	Present results of all sensitivity analyses conducted to assess the robustness of the synthesized results.	NA
Reporting biases	21	Present assessments of risk of bias due to missing results (arising from reporting biases) for each synthesis assessed.	NA
Certainty of evidence	22	Present assessments of certainty (or confidence) in the body of evidence for each outcome assessed.	NA
**DISCUSSION**			
Discussion	23a	Provide a general interpretation of the results in the context of other evidence.	7-9
	23b	Discuss any limitations of the evidence included in the review.	9-10
	23c	Discuss any limitations of the review processes used.	9-10
	23d	Discuss implications of the results for practice, policy, and future research.	10
**OTHER INFORMATION**			
Registration and protocol	24a	Provide registration information for the review, including register name and registration number, or state that the review was not registered.	4
	24b	Indicate where the review protocol can be accessed, or state that a protocol was not prepared.	4
	24c	Describe and explain any amendments to information provided at registration or in the protocol.	NA
Support	25	Describe sources of financial or non-financial support for the review, and the role of the funders or sponsors in the review.	10-11
Competing interests	26	Declare any competing interests of review authors.	10
Availability of data, code and other materials	27	Report which of the following are publicly available and where they can be found: template data collection forms; data extracted from included studies; data used for all analyses; analytic code; any other materials used in the review.	11

From: Page MJ, McKenzie JE, Bossuyt PM, Boutron I, Hoffmann TC, Mulrow CD, et al. The PRISMA 2020 statement: an updated guideline for reporting systematic reviews. BMJ 2021;372:n71. doi: 10.1136/bmj.n71

eTable 3. Characteristics of 76 original articles providing data on the use of heated tobacco products (HTP) included in the review, with corresponding information contributing to the systematic review

First author, year	Countries	Year of data collection	Sample size	Population	Current HTP use	Ever HTP use	Dual use	Use transitions	Strata
Adamson et al, 2020[1]	Japan	2018	4,156	Age 20+ living in Tokyo, Osaka and Sendai	X	X	X	X	
AlMulla et al, 2021 [2]	Qatar	2019	6,904	University students and governmental employees	X		X		Smoking status
Arslan et al, 2020 [3]	Turkey	2019	332	Family physicians working in Samsun	X	X			
Atzendorf et al, 2019[4]	Germany	2018	9,267	Adults	X				Sex
Azagba et al, 2021 [5]	USA	2019	42,477	Civilian non-institutionalized adults		X			
Berg et al, 2021 [6]	USA	2018	2,375	Young adults between 18 and 34 years of age		X			
Brose et al, 2018 [7]	UK	2017	12,693	Age 17+	X	X	X		Sex, age, SES, smoking status
Brose et al, 2021 [8]	UK	2019	3,883	Adult former or current smokers or vapers	X	X			
Cerrai et al, 2020 [9]	Italy	2018	15,732	Students between 15 and 19 years of age	X	X			

Chang et al, 2020 [10]	Taiwan	2018	44,905	Adolescents between 12 and 18 years of age	X	X			
Chung et al, 2020 [11]	South Korea	2018	60,040	Adolescents between 13 and 18 years of age		X			
Cox et al, 2021 [12]	UK	2020	8,486	Age 16+		X	X		
Cruz-Jiménez et al, 2022 [13]	Mexico	2021	6,500	Current smokers and/or vapers	X	X			
Dunbar et al, 2020 [14]	USA	2019	2,697	Young adults in south California (mean age=21.6)	X	X			
East et al, 2021 [15]	Canada, UK and USA	2018 and 2019	11,753 (2018) and 11,609 (2019)	Adolescents between 16 and 19 years of age	X				
Gallus et al, 2021 [16]	Italy	2019	3,120	Age 15+	X	X	X	X	Sex, age
Gallus et al, 2022 [17]	Bulgaria, France, Germany, Greece, Italy, Latvia, Poland, Portugal, Romania, Spain and UK	2017	10,961	Age 15+	X	X	X		Sex, age, SES, smoking status
Gallus et al, 2022 [18]	Italy	2020	6,003	Adults	X				
Gallus et al, 2022 [19]	Italy	2020	3,185	Adults				X	
Gentzke et al, 2020 [20]	USA	2020	14,531	Middle and high school students	X				
Gottschlich et al, 2020 [21]	Guatemala	2019	2,870	Students between 13 and 17 years of age attending Guatemala City private schools	X	X			
Gravely et al, 2020 [22]	Japan	2018	3,838	Smokers aged 20+	X				
Han et al, 2021 [23]	South Korea	2019	57,303	Middle and high school students	X	X			

Harada et al, 2022 [24]	Japan	2019	3,334	Resident population and worksite population in the Yamagata Prefecture	X		X		Sex, age
Havermans et al, 2021 [25]	Netherlands	2020	5,805	Age 13+	X	X	X		Sex, age, SES, smoking status
Hirai et al, 2021 [26]	Japan	2020	441	COPD patients with smoking history aged 40+ in four prefectures	X				
Huh et al, 2021 [27]	South Korea	2019	57,069	Middle and high school students	X				
Hwang et al, 2019 [28]	South Korea	2018	21,100	Age 19+ living in Kyungpook province	X	X	X		Sex, age, SES, smoking status
Hwang et al, 2020 [29]	South Korea	2019	57,303	Middle and high school students	X	X			
Hwang et al, 2021 [30]	South Korea	2018	1,200	Adults living in apartment in seven metropolitan cities		X			
Jankowski et al, 2019[31]	Poland	2018	423	Physicians attending mandatory public health courses	X	X			Sex
Jankowski et al, 2021[32]	Poland	2020	5,082	Police employees from the Mazowieckie province	X		X		Sex, age, smoking status
Kanai et al, 2021 [33]	Japan	2018-2019	158	Tobacco users at a manufacturing company				X	
Kang et al, 2020 [34]	South Korea	2018	59,532	Adolescents between 12 and 18 years of age		X			
Kang et al, 2021 [35]	South Korea	2018	60,040	Middle and high school students		X			
Kim et al, 2020 [36]	South Korea	2018	6,182	Age 19+	X		X		Sex, age, SES, smoking status

Kim et al, 2021 [37]	South Korea	2018	7,000	Age 20+	X	X	X		Sex, SES, smoking status
Kinjo et al, 2020 [38]	Japan	2018	4,628	Age 20+	X	X			Sex, age, SES
Kioi et al, 2018 [39]	Japan	2015	4,432	Age 40+	X	X	X		Sex, age
Koyama et al, 2021 [40]	Japan	2020	5,120	Tobacco users				X	
Kuwabara et al, 2020[41]	Japan	2017	64,152	Middle and high school students (mean age=15.7)	X	X			
Kwon et al, 2021 [42]	South Korea	2019	4,028	Smokers between 12 and 18 years of age	X				
Laverty et al, 2021 [43]	27 European countries and UK	2020	27,786	Age 15+	X	X	X		Sex, age, smoking status
Lee et al, 2019 [44]	South Korea	2018	60,040	Adolescents between 12 and 18 years of age		X			
Lee et al, 2021 [45]	South Korea	2020	663	Female smokers aged 19+	X				
Lee et al, 2021 [46]	South Korea	2018	7,000	Age 20+	X				Age
Lee et al, 2021 [47]	USA	2019	19,018	Middle and high school students	X	X			
Li et al, 2021 [48]	Australia, Canada, UK and USA	2020	10,296	Adult former and current cigarette smokers	X				
Li et al, 2021 [49]	USA	2020	150,516	Californian high school students	X	X			
Liu et al, 2019 [50]	Italy	2017	3,086	Age 15+		X	X		
Lotrean et al, 2020 [51]	Germany, Greece, Hungary, Poland, Romania and Spain	2016 and 2018	6,011 (2016) and 6,027 (2018)	Adult smokers	X	X			
Luk et al, 2021 [52]	Hong Kong	2018	1,213	Daily smokers				X	
Majek et al, 2021 [53]	Poland	2019	1,344	Medical students at the Medical	X	X			

				University of Silesia in Katowice					
Marynak et al, 2018[54]	USA	2017	4,107	Adult population		X	X		
Matsuyama et al, 2022 [55]	Japan	2019 and 2020	7,766 (2019) and 5,947 (2020)	Adult non-smokers of conventional cigarettes				X	
McKelvey et al, 2021[56]	USA	2018	450	Californian students (mean age=19.3)		X			
Miller et al, 2021 [57]	Australia, Canada, UK and USA	2018	11,421	Adult smokers	X				
Miller et al, 2022 [58]	Australia, Canada, UK and USA	2018	12,987	Adult former and current smokers	X	X			
Myagmar-Ochir et al, 2021 [59]	Japan	2018	7,714	Retail business workers	X		X		Sex
Nyman et al, 2018 [60]	USA	2016 and 2017	6,014 (2016) and 5,992 (2017)	Adult population	X	X	X		Sex, age, SES, smoking status
Odani et al, 2022 [61]	Japan	2020	9,044	Age 15+	X		X		Sex, age, smoking status
Park et al, 2021 [62]	South Korea	2019	57,303	Middle and high school students	X	X	X		
Park et al, 2022 [63]	South Korea	2018	2,000	Tobacco product users aged 19+	X	X			
Pinkas et al, 2019 [64]	Poland	2019	1,011	Age 15+	X				Sex
Pokhrel et al, 2021 [65]	USA	na	2,229	Hawaiian university students	X	X			
Sansone et al, 2020[66]	Japan	2018	4,684	Smokers aged 20+	X				
Sugiyama et al, 2020[67]	Japan	2017	10,114	Age 15+	X		X		
Sutanto et al, 2019 [68]	Japan	2018	4,684	Age 20+	X		X		
Tabuchi et al, 2016 [69]	Japan	2015	8,240	Age 15+		X	X		
Tabuchi et al, 2018 [70]	Japan	2015, 2016 and 2017	8,240	Age 15+	X		X		Sex, age, SES, smoking status

Tattan-Birch et al, 2021 [71]	UK	2016 and 2020	75,355	Age 16+	X		X		Sex, age, SES
Wang et al, 2021 [72]	Hong Kong	2019	33,991	High school students	X	X			
Wu et al, 2020 [73]	Hong Kong	2017	5,131	Chinese population aged 15+		X	X		
Xia et al, 2022 [74]	Hong Kong	2016-2019	579	Young smokers who want to quit				X	
Yi et al, 2021 [75]	South Korea	2017	271	Male current or former smokers	X				
Zhu et al, 2021 [76]	USA	2020	20,449	Adult population	X	X			

COPD, Chronic obstructive pulmonary disease; SES, socio-economic status.

eTable 4. Prevalence of use of heated tobacco products (HTP) among adults by country

Country	First Author, year	Year of conduction	Ever HTP use (%)	Current HTP use (%)
Austria	Laverty et al, 2021 [43]	2020	12.0	2.0
Belgium	Laverty et al, 2021 [43]	2020	7.8	1.7
Bulgaria	Gallus et al, 2022 [17]	2017	2.0	0.3
	Laverty et al, 2021 [43]	2020	12.0	2.4
Croatia	Laverty et al, 2021 [43]	2020	6.8	0.7
Cyprus	Laverty et al, 2021 [43]	2020	8.2	3.1
Czech Republic	Laverty et al, 2021 [43]	2020	14.6	3.1
Denmark	Laverty et al, 2021 [43]	2020	6.0	0.3
Estonia	Laverty et al, 2021 [43]	2020	8.2	1.1
Finland	Laverty et al, 2021 [43]	2020	9.5	1.3
France	Gallus et al, 2022 [17]	2017	1.8	0.2
	Laverty et al, 2021 [43]	2020	2.8	0.8
Germany	Atzendorf et al, 2019 [4]	2018	-	0.8
	Gallus et al, 2022 [17]	2018	1.4	0.1
	Laverty et al, 2021 [43]	2020	5.5	0.6
Greece	Gallus et al, 2022 [17]	2018	8.3	1.0
	Laverty et al, 2021 [43]	2020	9.0	1.9
Hong Kong	Wu et al, 2020 [73]	2017	1.0	-
Hungary	Laverty et al, 2021 [43]	2020	4.8	1.3
Ireland	Laverty et al, 2021 [43]	2020	12.3	1.8
Italy	Gallus et al, 2022 [17]	2016	1.1	0.0
	Liu et al, 2019 [50]	2017	1.4	-
	Gallus et al, 2021 [16]	2019	1.6	1.1
	Gallus et al, 2022 [18]	2020 (pre-lockdown)	-	4.0
	Gallus et al, 2022 [18]	2020 (post-lockdown)	-	4.5
	Laverty et al, 2021 [43]	2020	9.7	3.0
Japan	Tabuchi et al, 2018 [70]	2015	-	0.3
	Tabuchi et al, 2018 [70]	2016	-	0.6
	Tabuchi et al, 2018 [70]	2017	-	3.6
	Kioi et al, 2018 [39]	2015	0.1	0.0
	Tabuchi et al, 2016 [69]	2015	0.6	-
	Sugiyama et al, 2020 [67]	2017	-	2.7
	Kinjo et al, 2020 [38]	2018	8.7	5.0
	Sutanto et al, 2019 [68]	2018	-	2.7
	Adamson et al, 2020 [1]	2018	9.6	5.0
	Myagmar-Ochir et al, 2021 [59]	2018	-	6.4
	Odani et al, 2021 [61]	2020	-	10.9
Latvia	Gallus et al, 2022 [17]	2018	1.8	0.0
	Laverty et al, 2021 [43]	2020	13.8	2.9
Lithuania	Laverty et al, 2021 [43]	2020	10.6	2.2
Luxembourg	Laverty et al, 2021 [43]	2020	11.4	0.6
Malta	Laverty et al, 2021 [43]	2020	3.9	1.8
Netherlands	Laverty et al, 2021 [43]	2020	4.1	0.5

	Havermans et al, 2021 [25]	2020	3.0	0.4
Poland	Gallus et al, 2022 [17]	2018	1.6	0.0
	Jankowski et al, 2019 [31]	2018	8.5	1.9
	Jankowski et al, 2021 [32]	2020	-	5.5
	Laverty et al, 2021 [43]	2020	3.8	1.0
Portugal	Gallus et al, 2022 [17]	2017	3.0	0.5
	Laverty et al, 2021 [43]	2020	7.9	1.0
Qatar	AlMulla et al, 2021 [2]	2019	-	0.7
Romania	Gallus et al, 2022 [17]	2017	3.0	0.0
	Laverty et al, 2021 [43]	2020	5.4	0.5
Slovakia	Laverty et al, 2021 [43]	2020	9.6	2.5
Slovenia	Laverty et al, 2021 [43]	2020	7.4	1.1
South Korea	Kim et al, 2020 [36]	2018	-	4.4
	Hwang et al, 2019 [28]	2018	3.5	2.1
	Hwang et al, 2021 [30]	2018	23.9	-
	Kim et al, 2021 [37]	2018	16.8	10.2
Spain	Gallus et al, 2022 [17]	2017	0.6	0.1
	Laverty et al, 2021 [43]	2020	6.3	1.0
Sweden	Laverty et al, 2021 [43]	2020	6.6	0.4
Turkey	Arslan et al, 2020 [3]	2019	2.7	0.3
UK	Tattan-Birch et al, 2021 [71]	2016	-	0.2
	Gallus et al, 2022 [17]	2017	2.1	0.2
	Brose et al, 2018 [7]	2017	1.8	0.8
	Cox et al, 2021 [12]	2020	0.1	-
	Laverty et al, 2021 [43]	2020	6.6	0.9
	Tattan-Birch et al, 2021 [71]	2020	-	0.2
USA	Nyman et al, 2018 [60]	201	1.4	0.5
	Nyman et al, 2018 [60]	2017	2.2	1.1
	Marynak et al, 2018 [54]	2017	0.7	-
	Azagba et al, 2021 [5]	2019	0.5	-
	Zhu et al, 2021 [76]	2020	0.6	0.1

HTP, heated tobacco products.

eTable 5. Prevalence of use of heated tobacco products (HTP) among teenagers by country

Country	First author, year	Year of conduction	Age (years)	Ever HTP use (%)	Current HTP use (%)
Canada	East et al. 2021 [15]	2018	16-19	-	0.6
	East et al. 2021 [15]	2019	16-19	-	1.0
Guatemala	Gottschilich et al. 2020 [21]	2019	13-17	11.3	2.9
Hong Kong	Wang et al. 2021 [72]	2018-2019	14.0 (mean)	2.6	1.6
Italy	Cerrai et al. 2020 [9]	2018	15-19	5.0	2.0
Japan	Kuwabara et al. 2020 [41]	2017-2018	12-18	1.8	0.8
South Korea	Chung et al. 2020 [11]	2018	13-18	2.9	-
	Kang et al. 2020 [34]	2018	12-18	2.8	-
	Kang et al, 2021 [35]	2018	12-18	2.9	-
	Lee et al, 2019 [44]	2018	12-18	2.9	-
	Han et al. 2021 [23]	2019	12-18	4.9	2.6
	Huh et al. 2021 [27]	2019	12-18	-	2.6
	Park et al. 2021 [62]	2019	12-18	4.7	2.4
	Hwang et al. 2020 [29]	2019	12-18	4.9	2.6
Taiwan	Chang et al, 2020 [10]	2018	12-18	4.2	2.3
UK	East et al. 2021 [15]	2018	16-19	-	0.6
	East et al. 2021 [15]	2019	16-19	-	0.8
USA	East et al. 2021 [15]	2018	16-19	-	1.3
	East et al. 2021 [15]	2019	16-19	-	1.4
	Lee et al. 2021 [47]	2019	11-18	2.3	1.6
	Li et al. 2021 [49]	2019-2020	14-18	0.7	0.2
	Gentzke et al. 2020 [20]	2020	11-18	-	1.4

HTP, heated tobacco products.

eTable 6. Quality evaluation of the 5 cohort studies included in the meta-analysis on use transitions using the New-Castle Ottawa (NOS) scale

**Author, Year**	**SELECTION**				**COMPARABILITY**	**EXPOSURE**			**TOTAL NOS SCORE**
	Representativeness of the exposed cohort	Selection of the non-exposed cohort	Ascertainment of exposure	Outcome of interest not present at start of study	Comparability of cohorts[a,b]	Ascertainment of outcome	Follow-up long enough for outcome to occur[c]	Adequacy of follow-up cohorts[d]	
Kanai, 2021 [33]	-	☆	-	-	☆☆	-	-	☆	4
Luk, 2021 [52]	-	☆	-	☆	☆☆	-	☆	-	5
Gallus, 2022 [19]	-	☆	-	-	☆	-	☆	-	3
Matsuyama, 2022 [55]	-	☆	-	-	☆	-	☆	-	3
Xia, 2022 [74]	-	☆	-	-	☆☆	-	☆	-	4

[a] Each item could be scored with a maximum of one star, except for the item "Comparability of cohorts" which could receive a maximum of two stars.

[b] Studies controlling for age or sex in the design or in the analysis received one star. Studies with all the previous variables and at least one of the following variables: previous quit attempt, pharmacological support, intention to quit, level of nicotine dependency received two stars.

[c] Studies with follow-up time ≥6 months received one star.

[d] Studies with follow-up rate ≥80% or with a description of those lost at follow-up received one star.

eTable 7. Quality evaluation of the 21 cross-sectional studies providing results on the association between heated tobacco products use and socio-demographic characteristics (sex, age, socio-economic status, use of conventional cigarette) using the New-Castle Ottawa scale adapted for cross-sectional studies

**Author, Year**	**SELECTION**			**COMPARABILITY**	**OUTCOME**		**TOTAL NOS SCORE**	Socio-demographic characteristics evaluated
	Representativeness of the sample	Non-respondents	Ascertainment of the exposure	Comparability[a,b]	Ascertainment of outcome	Statistical test		
AlMulla, 2021[2]	☆	☆	-	-	-	-	2	Smoke
Atzendorf, 2019[4]	☆	-	-	-	-	-	1	Sex
Brose, 2018[7]	☆	-	-	-	-	-	1	Sex, age, smoke
Gallus, 2021[16]	☆	-	-	☆☆	-	☆	4	Sex, age
Gallus, 2022[17]	☆	☆	-	☆☆	-	☆	5	Sex, age, SES, smoke
Harada, 2022[24]	-	-	-	-	-	-	0	Sex, age
Havermans, 2021[25]	☆	-	-	-	-	-	1	Sex, age, SES, smoke
Hwang, 2019[28]	☆	☆	-	☆☆	-	☆	5	Sex, age, SES, smoke
Jankowski, 2019[31]	-	☆	-	-	-	-	1	Sex
Jankowski, 2021[32]	-	-	-	-	-	-	0	Sex, age, smoke
Kim, 2020[36]	☆	-	-	☆☆	-	☆	4	Sex, age, SES, smoke
Kim, 2021[37]	☆	-	-	☆☆	-	☆	4	Sex, SES, smoke
Kinjo, 2020[38]	☆	-	-	-	-	-	1	Sex, age, SES
Kioi, 2018[39]	-	-	-	-	-	-	0	Sex
Laverty, 2021[43]	☆	☆	-	☆☆	-	☆	5	Sex, age, smoke
Myagmar-Ochir, 2021[59]	-	☆	-	-	-	-	1	Sex
Nyman, 2018[60]	☆	-	-	☆☆	-	☆	4	Sex, age, SES, smoke
Odani, 2022[61]	☆	-	-	-	-	-	1	Sex, age, smoke

Pinkas, 2019[64]	☆	-	-	-	-	-	1	Sex
Tabuchi, 2018[70]	☆	-	-	☆☆	-	☆	4	Sex, age, SES, smoke
Tattan-Birch, 2021[71]	☆	-	-	-	-	-	1	Sex, age

NOS, New-Castle Ottawa Scale; SES, socioeconomic status.

[a] Each item could be scored with a maximum of one star, except for the item "Comparability of cohorts" which could receive a maximum of two stars.

[b] Studies controlling for age or sex in the design or in the analysis received one star. Studies with all the previous variables and at least one of the following variables: level of education, income, cigarette use, e-cigarette use, alcohol consumption received two stars.

eFigure 1. Flowchart of the systematic review

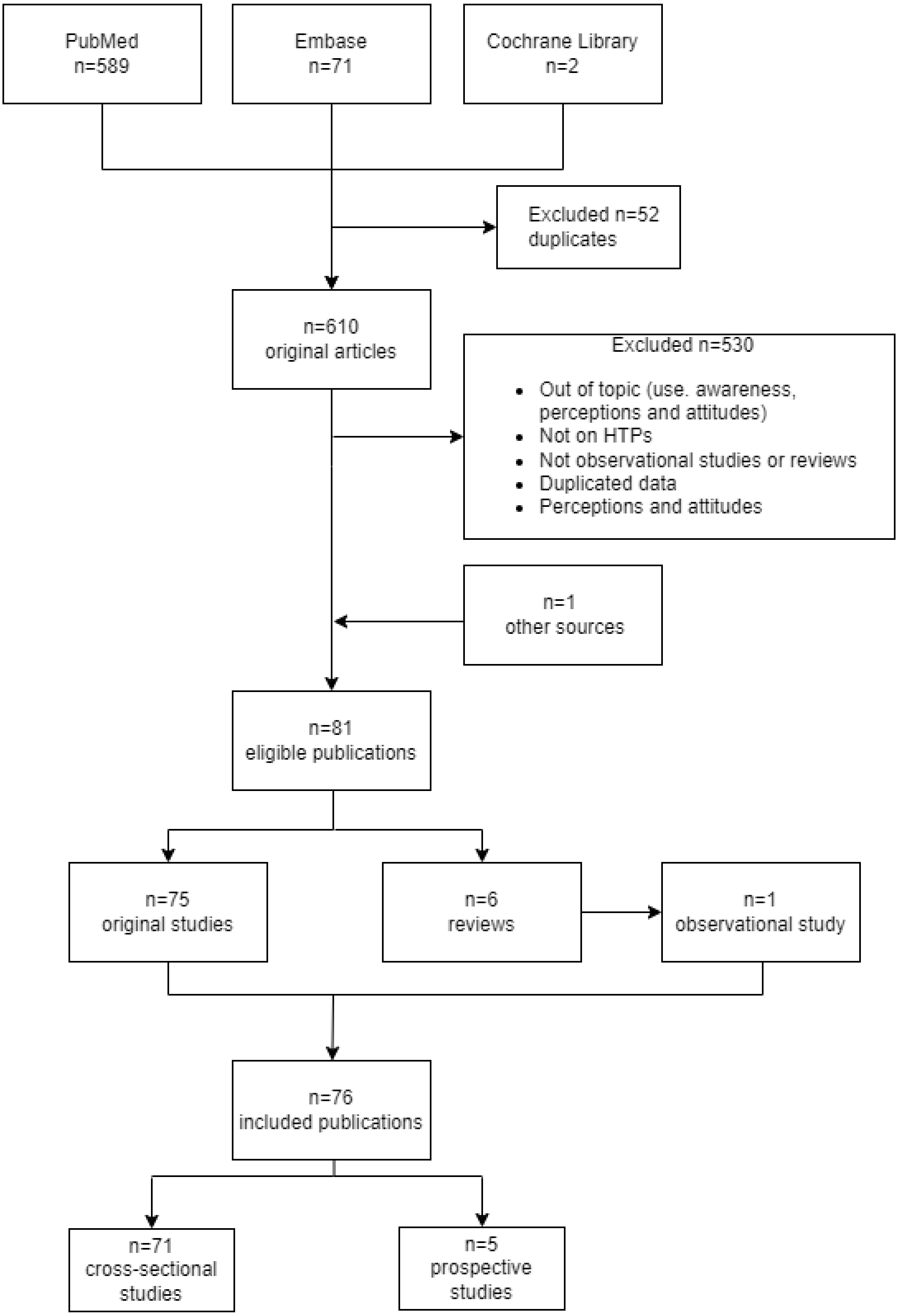

eFigure 2. Forest plot of pooled odds ratio for current heated tobacco product use among adults according to sex (male vs female), overall and stratified by continent

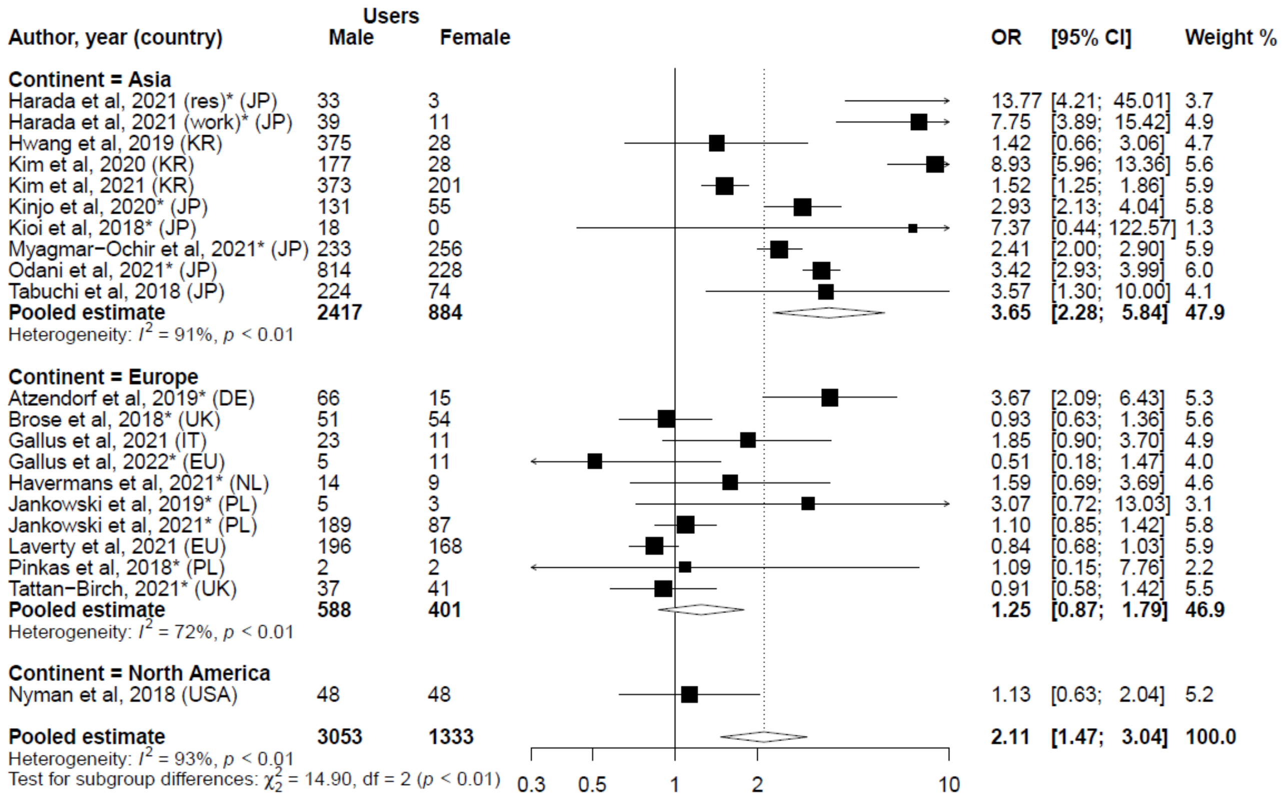

*OR values estimated from raw data. CI, confidence interval; DE, Germany; EU, Europe (different countries); IT, Italy; JP, Japan; KR, South Korea; NL, Netherlands; OR, odds ratio; PL, Poland; res, resident population; UK, United Kingdom; USA, United States of America; work, working population.

eFigure 3. Forest plot of pooled odds ratios for current heated tobacco product use among adults according to age group (middle aged vs young adults), overall and stratified by continent

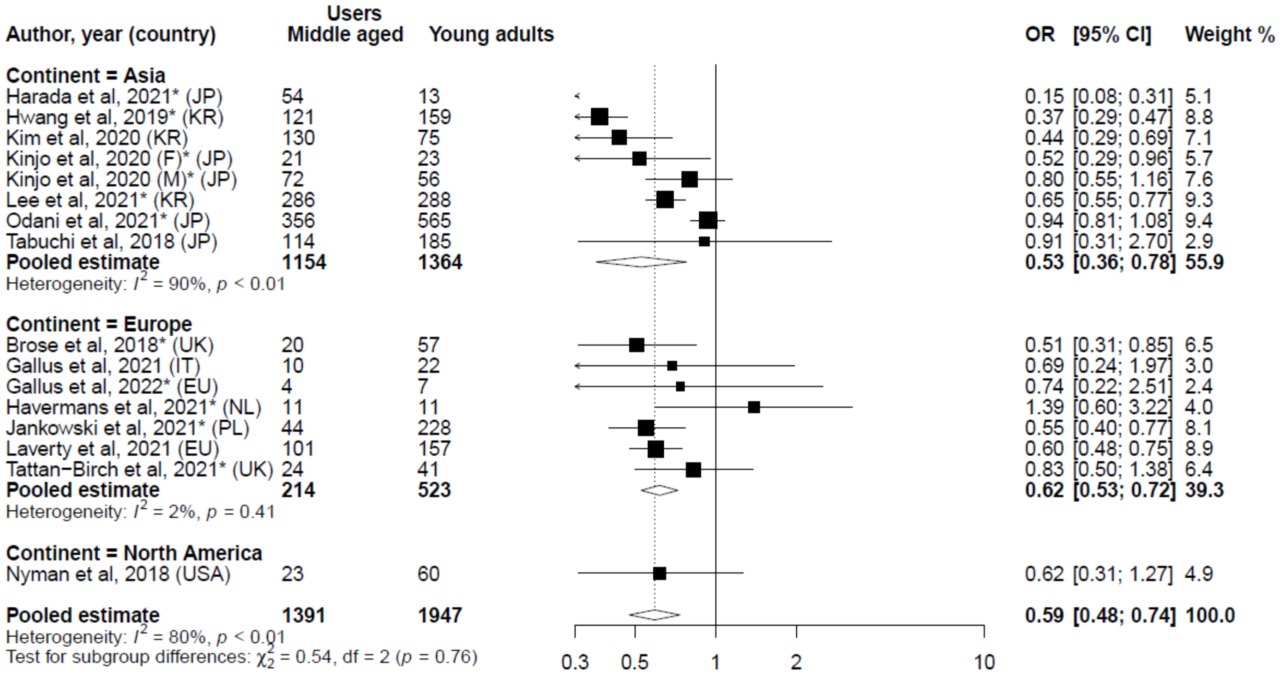

*OR values estimated from raw data. CI, confidence interval; EU, Europe (different countries); F, female; IT, Italy, JP, Japan; KR, South Korea; M, male; NL, Netherlands; OR, odds ratio; PL, Poland; UK, United Kingdom; USA, United States of America.

eFigure 4. Forest plot of pooled odds ratios for current heated tobacco product use among adults according to age group (old adults vs young adults), overall and stratified by continent

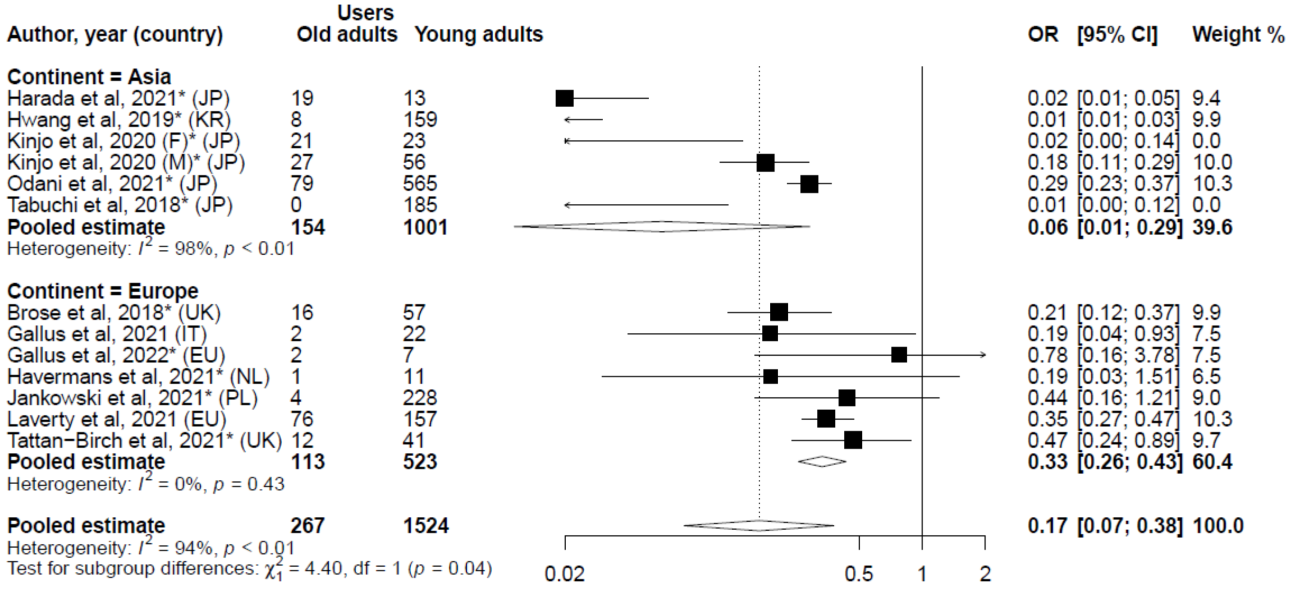

*OR values estimated from raw data. CI, confidence interval; EU, Europe (different countries); F, female; IT, Italy; JP, Japan; KR, South Korea; M, male; NL, Netherlands; OR, odds ratio; PL, Poland, UK, United Kingdom.

eFigure 5. Forest plot of pooled odds ratios for current heated tobacco product use among adults according to socio-economic status (intermediate vs low), overall and stratified by continent

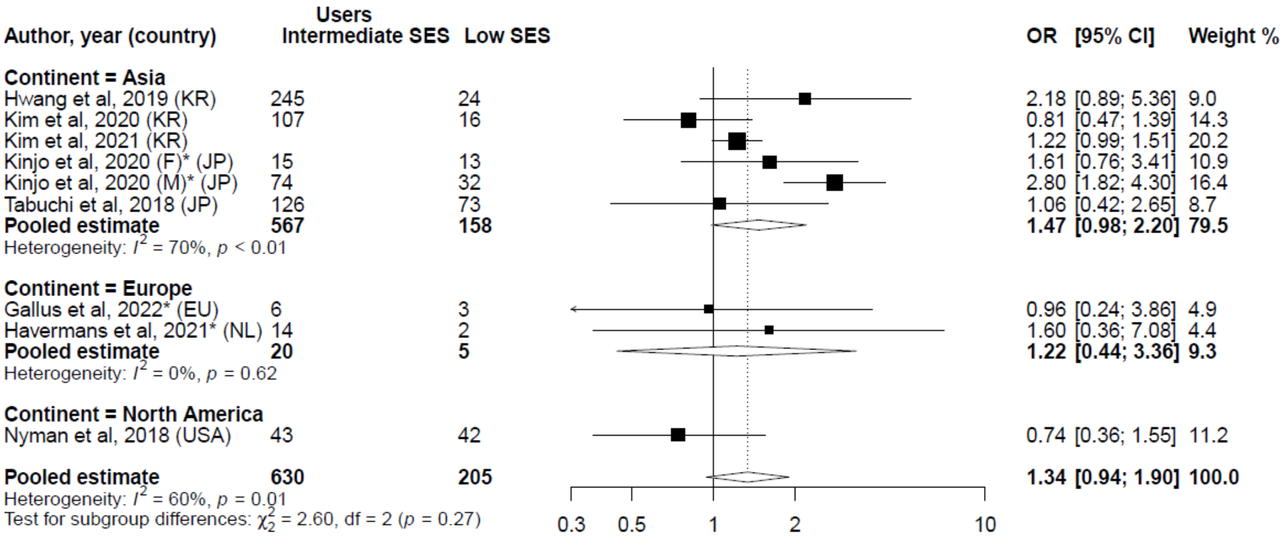

*****OR values estimated from raw data. CI, confidence interval; EU, Europe (different countries); F, female; JP, Japan; KR, South Korea; M, male; NL, Netherlands; OR, odds ratio; SES, socioeconomic status; USA, United States of America.

eFigure 6. Forest plot of pooled odds ratios for current heated tobacco product use among adults according to socio-economic status (high vs low), overall and stratified by continent

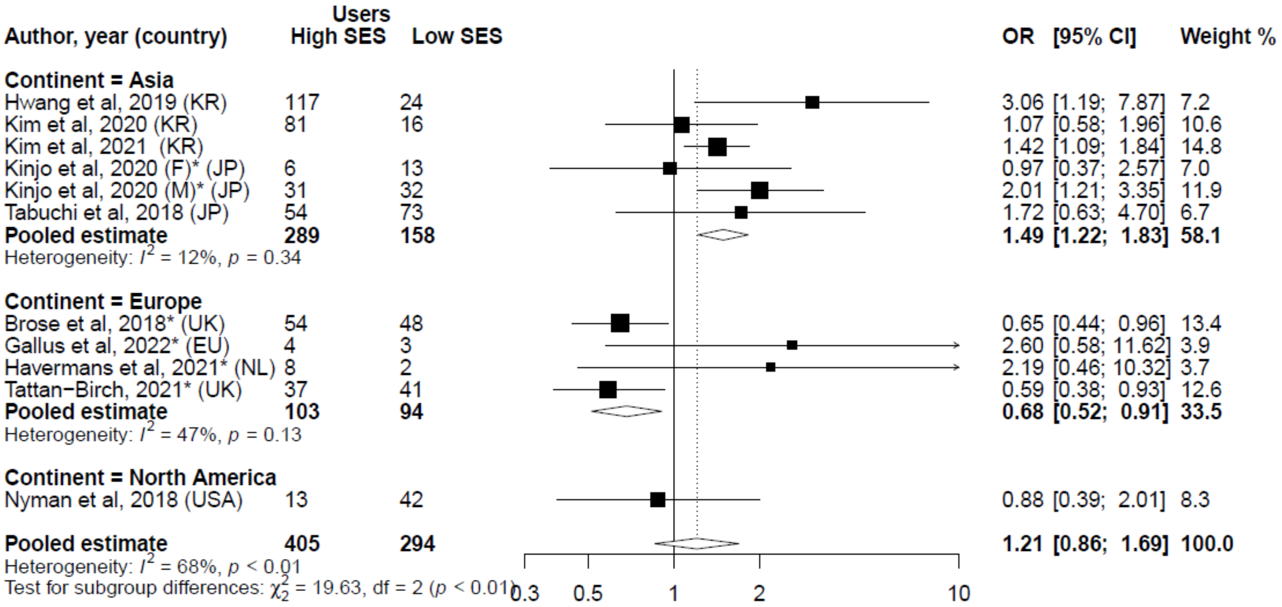

*OR values estimated from raw data. CI, confidence interval; F, female; EU, Europe (different countries); JP, Japan; KR, South Korea; M, male; NL, Netherlands; OR, odds ratio; UK, United Kingdom; SES, socioeconomic status; USA, United States of America.

eFigure 7. Forest plot of pooled odds ratios for current heated tobacco product use among adults according to cigarette smoking status (former vs never smokers), overall and stratified by continent

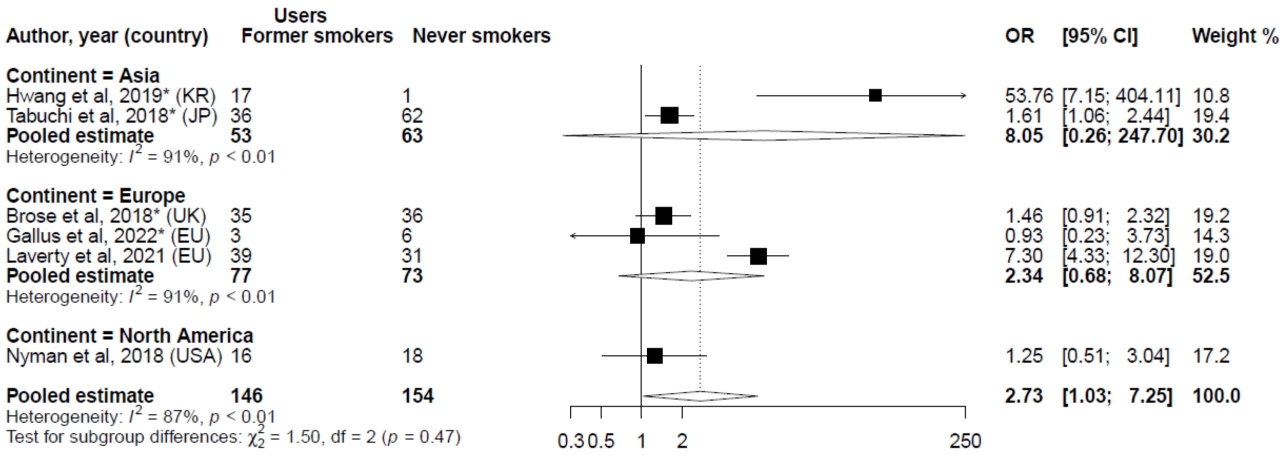

*OR values estimated from raw data. CI, confidence interval; EU, Europe (different countries); JP, Japan; KR, South Korea; OR, odds ratio; UK, United Kingdom; USA, United States of America.

eFigure 8. Forest plot of pooled odds ratios for current heated tobacco product use among adults according to cigarette smoking status (current vs non/never smokers), overall and stratified by continent

Author, year (country)	Users: Current smokers	Users: Never/non smokers	OR	[95% CI]	Weight %
**Continent = Asia**					
AlMulla et al, 2021 * (QA)	32	15	12.51	[6.75; 23.18]	8.6
Hwang et al, 2019* (KR)	339	1	1356.51	[190.46; 9661.40]	6.0
Kim et al, 2020 (KR)	170	35	25.67	[17.72; 37.18]	8.8
Kim et al, 2021* (KR)	464	110	25.90	[20.80; 32.26]	8.9
Odani et al, 2021* (JP)	778	208	15.53	[13.20; 18.27]	9.0
Tabuchi et al, 2018* (JP)	200	62	9.88	[7.39; 13.21]	8.9
**Pooled estimate**	**1983**	**431**	**27.64**	**[9.17; 83.31]**	**50.2**
Heterogeneity: I^2 = 91%, $p < 0.01$					
**Continent = Europe**					
Brose et al, 2018* (UK)	32	36	3.66	[2.27; 5.91]	8.7
Gallus et al, 2022* (EU)	9	6	2.80	[1.00; 7.88]	7.9
Havermans et al, 2021* (NL)	22	2	47.66	[11.19; 203.00]	7.1
Jankowski et al, 2021* (PL)	200	59	7.73	[5.75; 10.40]	8.9
Laverty et al, 2021 (EU)	280	31	36.30	[22.92; 57.49]	8.8
**Pooled estimate**	**543**	**134**	**10.19**	**[3.42; 30.36]**	**41.3**
Heterogeneity: I^2 = 93%, $p < 0.01$					
**Continent = North America**					
Nyman et al, 2018 (USA)	79	18	1.57	[0.81; 3.04]	8.5
**Pooled estimate**	**2605**	**583**	**14.53**	**[6.34; 33.31]**	**100.0**
Heterogeneity: I^2 = 94%, $p < 0.01$					
Test for subgroup differences: χ^2_2 = 22.23, df = 2 ($p < 0.01$)					

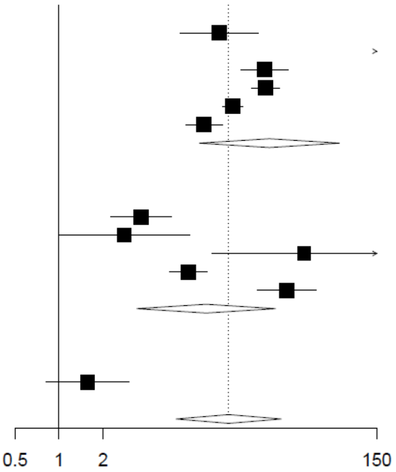

*OR values estimated from raw data. CI, confidence interval; EU, Europe (different countries), JP, Japan; KR, South Korea; NL, Netherlands; OR, odds ratio; PL, Poland; QA, Qatar; UK, United Kingdom; USA, United States of America.

eFigure 9. Forest plot of pooled odds ratios for conventional cigarette smoking initiation among never smokers according to heated tobacco product use (current vs non/never users)

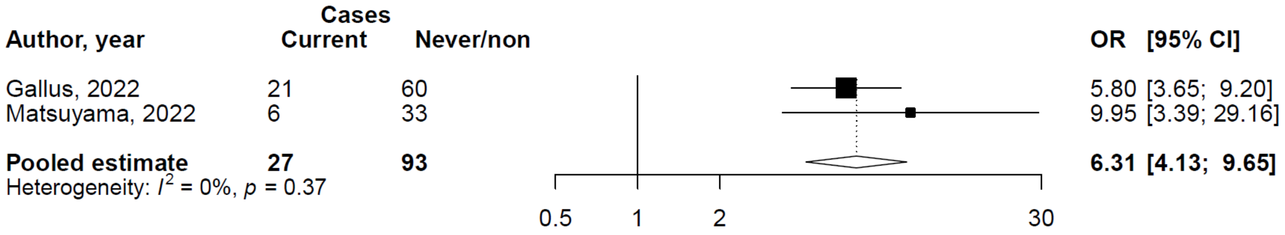

CI, confidence interval; OR, odds ratio.

eFigure 10. Forest plot of pooled odds ratios for conventional cigarette smoking relapse among former smokers according to heated tobacco product use (current vs non/never users)

Author, year	Cases Current	Cases Never/non	OR [95% CI]
Gallus, 2022	14	31	3.32 [2.05; 5.37]
Matsuyama, 2022a	19	25	0.59 [0.28; 1.26]
Matsuyama, 2022b	14	39	2.80 [1.42; 5.52]
Xia, 2022	17	62	4.56 [1.17; 17.79]
**Pooled estimate**	**64**	**157**	**2.15 [0.89; 5.21]**

Heterogeneity: $I^2 = 81\%$, $p < 0.01$

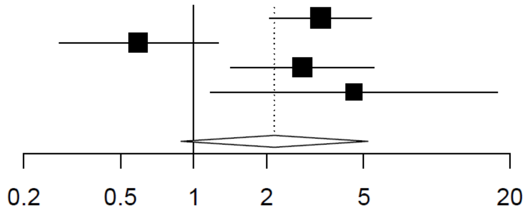

CI, confidence interval; OR, odds ratio.

eFigure 11. Forest plot of pooled odds ratios for conventional cigarette smoking cessation among current smokers according to heated tobacco product use (current vs non/never users)

**Author, year**	**Cases Current**	**Cases Never/non**	**OR**	**[95% CI]**
Gallus, 2022	0	100	0.85	[0.81; 0.91]
Kanai, 2021	16	29	0.77	[0.61; 0.97]
Luk, 2021	15	263	1.08	[0.63; 1.85]
Xia, 2022	15	120	0.47	[0.24; 0.91]
**Pooled estimate**	**46**	**512**	**0.84**	**[0.80; 0.89]**

Heterogeneity: I^2 = 32%, p = 0.22

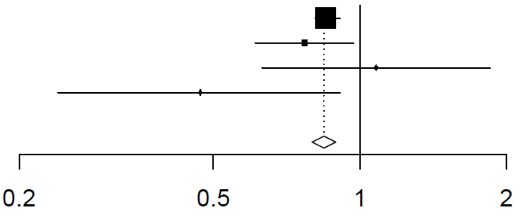

CI, confidence interval; OR, odds ratio.

REFERENCES

1. Adamson J, Kanitscheider C, Prasad K, et al. Results from a 2018 cross-sectional survey in Tokyo, Osaka and Sendai to assess tobacco and nicotine product usage after the introduction of heated tobacco products (HTPs) in Japan. Harm Reduction Journal 2020; 17: 32.

2. AlMulla A, Mamtani R, Cheema S, et al. Epidemiology of tobacco use in Qatar: Prevalence and its associated factors. PLOS ONE 2021; 16: e0250065.

3. Arslan HN, Oruc MA, Terzi O, Bilir N. Evaluation of the Opinions of Family Physicians on Some Tobacco Products. Journal of Community Health 2020; 45: 1132-1138.

4. Atzendorf J, Rauschert C, Seitz NN, et al. The Use of Alcohol, Tobacco, Illegal Drugs and Medicines. Dtsch Arztebl Int 2019; 116: 577-584.

5. Azagba S, Shan L. Heated Tobacco Products: Awareness and Ever Use Among U.S. Adults. American Journal of Preventive Medicine 2021; 60: 684-691.

6. Berg CJ, Romm KF, Patterson B, Wysota CN. Heated Tobacco Product Awareness, Use, and Perceptions in a Sample of Young Adults in the United States. Nicotine & Tobacco Research 2021; 23: 1967-1971.

7. Brose L, Simonavicius E, Cheeseman H. Awareness and Use of 'Heat-not-burn' Tobacco Products in Great Britain. Tobacco Regulatory Science 2018; 4: 44-50.

8. Brose LS, McDermott MS, McNeill A. Heated Tobacco Products and Nicotine Pouches: A Survey of People with Experience of Smoking and/or Vaping in the UK. International Journal of Environmental Research and Public Health 2021; 18: 8852.

9. Cerrai S, Potente R, Gorini G, et al. What is the face of new nicotine users? 2012–2018 e-cigarettes and tobacco use among young students in Italy. International Journal of Drug Policy 2020; 86: 102941.

10. Chang L-C, Lee Y-C, Hsu C, Chen P-C. Prevalence of heated tobacco product use among adolescents in Taiwan. PLOS ONE 2020; 15: e0244218.

11. Chung SJ, Kim B-K, Oh JH, et al. Novel tobacco products including electronic cigarette and heated tobacco products increase risk of allergic rhinitis and asthma in adolescents: Analysis of Korean youth survey. Allergy 2020; 75: 1640-1648.

12. Cox S, Brown J, Kock L, Shahab L. Prevalence and characteristics of ever regular use of non-combustible nicotine for 1 year or more: a population survey in England. Harm Reduction Journal 2021; 18: 114.

13. Cruz-Jiménez L, Barrientos-Gutiérrez I, Zavala-Arciniega L, et al. Heated tobacco product use, its correlates, and reasons for use among Mexican smokers. Drug and Alcohol Dependence 2022; 232: 109283.

14. Dunbar MS, Seelam R, Tucker JS, et al. Correlates of Awareness and Use of Heated Tobacco Products in a Sample of US Young Adults in 2018–2019. Nicotine & Tobacco Research 2020; 22: 2178-2187.

15. East KA, Reid JL, Rynard VL, Hammond D. Trends and Patterns of Tobacco and Nicotine Product Use Among Youth in Canada, England, and the United States From 2017 to 2019. Journal of Adolescent Health 2021; 69: 447-456.

16. Gallus S, Borroni E, Odone A, et al. The Role of Novel (Tobacco) Products on Tobacco Control in Italy. International Journal of Environmental Research and Public Health 2021; 18: 1895.

17. Gallus S, Lugo A, Liu X, Borroni E, Clancy L, Gorini G, Lopez MJ, Odone A, Przewozniak K, Tigova O, van den Brandt PA, Vardavas C, Fernandez E; TackSHS Project Investigators. Use and Awareness of Heated Tobacco Products in Europe. Journal of Epidemiology 2022; 32: 139-144.

18. Gallus S, Stival C, Carreras G, et al. Use of electronic cigarettes and heated tobacco products during the Covid-19 pandemic. Scientific Reports 2022; 12: 702.

19. Gallus S, Stival C, McKee M, et al. Impact of electronic cigarette and heated tobacco product on conventional smoking: an Italian prospective cohort study conducted during the COVID-19 pandemic. Tob Control 2024;33(2):267-270.

20. Gentzke AS, Wang TW, Jamal A, et al. Tobacco Product Use Among Middle and High School Students - United States, 2020. MMWR Morb Mortal Wkly Rep 2020; 69: 1881-1888.

21. Gottschlich A, Mus S, Monzon JC, et al. Cross-sectional study on the awareness, susceptibility and use of heated tobacco products among adolescents in Guatemala City, Guatemala. BMJ Open 2020; 10: e039792.

22. Gravely S, Fong GT, Sutanto E, et al. Perceptions of Harmfulness of Heated Tobacco Products Compared to Combustible Cigarettes among Adult Smokers in Japan: Findings from the 2018 ITC Japan Survey. International Journal of Environmental Research and Public Health 2020; 17: 2394.

23. Han MA. Ability to Purchase Tobacco Products and Smoking Behavior of Cigarettes, E-cigarettes, and Heated Tobacco Products in Korean Adolescents. Am J Health Behav 2021; 45: 635-641.

24. Harada S, Sata M, Matsumoto M, et al. Changes in Smoking Habits and Behaviors Following the Introduction and Spread of Heated Tobacco Products in Japan and Its Effect on FEV₁ Decline: A Longitudinal Cohort Study. Journal of Epidemiology 2022; 32: 180-187.

25. Havermans A, Pennings JLA, Hegger I, et al. Awareness, use and perceptions of cigarillos, heated tobacco products and nicotine pouches: A survey among Dutch adolescents and adults. Drug and Alcohol Dependence 2021; 229: 109136.

26. Hirai K, Tanaka A, Homma T, et al. Characteristics of and reasons for patients with chronic obstructive pulmonary disease to continue smoking, quit smoking, and switch to heated tobacco products. Tobacco Induced Diseases 2021; 19: 85.

27. Huh Y, Cho H-J. Associations between the Type of Tobacco Products and Suicidal Behaviors: A Nationwide Population-Based Study among Korean Adolescents. International Journal of Environmental Research and Public Health 2021; 18: 367.

28. Hwang JH, Ryu DH, Park S-W. Heated tobacco products: Cigarette complements, not substitutes. Drug and Alcohol Dependence 2019; 204: 107576.

29. Hwang JH, Ryu DH, Park I, Park S-W. Cigarette or E-Cigarette Use as Strong Risk Factors for Heated Tobacco Product Use among Korean Adolescents. International Journal of Environmental Research and Public Health 2020; 17: 7005.

30. Hwang J, Cho SI. A comparative study on changes in the use of heat-not-burn tobacco products based on whether apartment buildings have designated non-smoking areas. Tob Prev Cessat 2021; 7: 46.

31. Jankowski M, Kaleta D, Zgliczyński WS, et al. Cigarette and E-Cigarette Use and Smoking Cessation Practices among Physicians in Poland. International Journal of Environmental Research and Public Health 2019; 16: 3595.

32. Jankowski M, Gujski M, Pinkas J, et al. The prevalence of cigarette smoking, e-cigarette use and heated tobacco use among police employees in Poland: a 2020 cross-sectional survey. International Journal of Occupational Medicine and Environmental Health 2021; 34: 629-645.

33. Kanai M, Kanai O, Tabuchi T, Mio T. Association of heated tobacco product use with tobacco use cessation in a Japanese workplace: a prospective study. Thorax 2021; 76: 615-617.

34. Kang H, Cho S-I. Heated tobacco product use among Korean adolescents. Tobacco Control 2020; 29: 466-468.

35. Kang SY, Lee S, Cho H-J. Prevalence and predictors of heated tobacco product use and its relationship with attempts to quit cigarette smoking among Korean adolescents. Tobacco Control 2021; 30: 192-198.

36. Kim SH, Cho HJ. Prevalence and correlates of current use of heated tobacco products among a nationally representative sample of Korean adults: Results from a cross-sectional study. Tob Induc Dis 2020; 18: 66.

37. Kim J, Lee S, Kimm H, et al. Heated tobacco product use and its relationship to quitting combustible cigarettes in Korean adults. PLOS ONE 2021; 16: e0251243.

38. Kinjo A, Kuwabara Y, Fujii M, et al. Heated Tobacco Product Smokers in Japan Identified by a Population-Based Survey. Journal of Epidemiology 2020; 30: 547-555.

39. Kioi Y, Tabuchi T. Electronic, heat-not-burn, and combustible cigarette use among chronic disease patients in Japan: A cross-sectional study. Tobacco Induced Diseases 2018; 16:41.

40. Koyama S, Tabuchi T, Okawa S, et al. Changes in Smoking Behavior Since the Declaration of the COVID-19 State of Emergency in Japan: A Cross-sectional Study From the Osaka Health App. J Epidemiol 2021; 31: 378-386.

41. Kuwabara Y, Kinjo A, Fujii M, et al. Heat-not-burn tobacco, electronic cigarettes, and combustible cigarette use among Japanese adolescents: a nationwide population survey 2017. BMC Public Health 2020; 20: 741.

42. Kwon M, Chung SJ, Lee J. Use of Single, Dual, and Poly Tobacco Products in Korean Adolescents. Asia Pacific Journal of Public Health 2021; 33: 571-578.

43. Laverty AA, Vardavas CI, Filippidis FT. Prevalence and reasons for use of Heated Tobacco Products (HTP) in Europe: an analysis of Eurobarometer data in 28 countries. The Lancet Regional Health - Europe 2021; 8: 100159.

44. Lee A, Lee K-S, Park H. Association of the Use of a Heated Tobacco Product with Perceived Stress, Physical Activity, and Internet Use in Korean Adolescents: A 2018 National Survey. International Journal of Environmental Research and Public Health 2019; 16: 965.

45. Lee A, Lee K-S, Lee D, et al. The Utilization of National Tobacco Cessation Services among Female Smokers and the Need for a Gender-Responsive Approach. International Journal of Environmental Research and Public Health 2021; 18: 5313.

46. Lee JA, Lee C, Cho H-J. Use of heated tobacco products where their use is prohibited. Tob Control 2023; 32(2): 146-152.

47. Lee J, Thompson LA, Salloum RG. Heated tobacco product use among US adolescents in 2019: The new tobacco risk. Tob Prev Cessat 2021; 7: 01.

48. Li L, Borland R, Cummings KM, et al. Patterns of Non-Cigarette Tobacco and Nicotine Use Among Current Cigarette Smokers and Recent Quitters: Findings From the 2020 ITC Four Country Smoking and Vaping Survey. Nicotine & Tobacco Research 2021; 23: 1611-1616.

49. Li S, Braden K, Zhuang Y-L, Zhu S-H. Adolescent Use of and Susceptibility to Heated Tobacco Products. Pediatrics 2021; 148(2):e2020049597.

50. Liu X, Lugo A, Spizzichino L, et al. Heat-not-burn tobacco products: concerns from the Italian experience. Tobacco Control 2019; 28: 113-114.

51. Lotrean LM, Trofor A, Radu-Loghin C, Eremia M, Mihaltan F, Driezen P, Kyriakos CN, Mons U, Demjén T, Fernández E, Katsaounou PA, Przewoźniak K, Filippidis FT, Gravely S, Fong GT, Vardavas CI; EUREST-PLUS Consortium. Awareness and use of heated tobacco products among adult smokers in six European countries: findings from the EUREST-PLUS ITC Europe Surveys. European Journal of Public Health 2020; 30(Suppl_3): iii78-iii83.

52. Luk TT, Weng X, Wu YS, et al. Association of heated tobacco product use with smoking cessation in Chinese cigarette smokers in Hong Kong: a prospective study. Tob Control 2021; 30: 653-659.

53. Majek P, Jankowski M, Nowak B, et al. The Frequency of Use and Harm Perception of Heated Tobacco Products (HTPs): The 2019 Cross-Sectional Survey among Medical Students from Poland. International Journal of Environmental Research and Public Health 2021; 18: 3381.

54. Marynak KL, Wang TW, King BA, et al. Awareness and Ever Use of "Heat-Not-Burn" Tobacco Products Among U.S. Adults, 2017. American Journal of Preventive Medicine 2018; 55: 551-554.

55. Matsuyama Y, Tabuchi T. Heated tobacco product use and combustible cigarette smoking relapse/initiation among former/never smokers in Japan: the JASTIS 2019 study with 1-year follow-up. Tobacco Control 2022; 31: 520-526.

56. McKelvey K, Baiocchi M, Halpern-Felsher B. PMI's heated tobacco products marketing claims of reduced risk and reduced exposure may entice youth to try and continue using these products. Tobacco Control 2020; 29(e1): e18-e24.

57. Miller CR, Sutanto E, Smith DM, et al. Characterizing Heated Tobacco Product Use Among Adult Cigarette Smokers and Nicotine Vaping Product Users in the 2018 ITC Four Country Smoking & Vaping Survey. Nicotine & Tobacco Research 2022; 24(4): 493-502.

58. Miller CR, Sutanto E, Smith DM, et al. Awareness, trial and use of heated tobacco products among adult cigarette smokers and e-cigarette users: findings from the 2018 ITC Four Country Smoking and Vaping Survey. Tobacco Control 2022; 31: 11-18.

59. Myagmar-Ochir E, Kaneko M, Tomiyama K, et al. Occupational difference in use of heated tobacco products: a cross-sectional analysis of retail workers in Japan. BMJ Open 2021; 11: e049395.

60. Nyman AL, Weaver SR, Popova L, et al. Awareness and use of heated tobacco products among US adults, 2016–2017. Tobacco Control 2018; 27(Suppl 1): s55-s61.

61. Odani S, Tabuchi T. Prevalence of heated tobacco product use in Japan: the 2020 JASTIS study. Tobacco Control 2022; 31: e64-e65.

62. Park S, Lee KS. Association of heated tobacco product use and secondhand smoke exposure with suicidal ideation, suicide plans and suicide attempts among Korean adolescents: A 2019 national survey. Tob Induc Dis 2021; 19: 72.

63. Park J, Kim HJ, Shin SH, et al. Perceptions of Heated Tobacco Products (HTPs) and Intention to Quit Among Adult Tobacco Users in Korea. Journal of Epidemiology 2022; 32: 357-362.

64. Pinkas J, Kaleta D, Zgliczyński WS, et al. The Prevalence of Tobacco and E-Cigarette Use in Poland: A 2019 Nationwide Cross-Sectional Survey. International Journal of Environmental Research and Public Health 2019; 16: 4820.

65. Pokhrel P, Herzog TA, Kawamoto CT, Fagan P. Heat-not-burn Tobacco Products and the Increased Risk for Poly-tobacco Use. Am J Health Behav 2021; 45: 195-204.

66. Sansone G, Fong GT, Meng G, et al. Secondhand Smoke Exposure in Public Places and Support for Smoke-Free Laws in Japan: Findings from the 2018 ITC Japan Survey. International Journal of Environmental Research and Public Health 2020; 17: 979.

67. Sugiyama T, Tabuchi T. Use of Multiple Tobacco and Tobacco-Like Products Including Heated Tobacco and E-Cigarettes in Japan: A Cross-Sectional Assessment of the 2017 JASTIS Study. International Journal of Environmental Research and Public Health 2020; 17: 2161.

68. Sutanto E, Miller C, Smith DM, et al. Prevalence, Use Behaviors, and Preferences among Users of Heated Tobacco Products: Findings from the 2018 ITC Japan Survey. International Journal of Environmental Research and Public Health 2019; 16: 4630.

69. Tabuchi T, Kiyohara K, Hoshino T, et al. Awareness and use of electronic cigarettes and heat-not-burn tobacco products in Japan. Addiction 2016; 111: 706-713.

70. Tabuchi T, Gallus S, Shinozaki T, et al. Heat-not-burn tobacco product use in Japan: its prevalence, predictors and perceived symptoms from exposure to secondhand heat-not-burn tobacco aerosol. Tobacco Control 2018; 27: e25-e33.

71. Tattan-Birch H, Brown J, Shahab L, Jackson SE. Trends in use of e-cigarette device types and heated tobacco products from 2016 to 2020 in England. Scientific Reports 2021; 11: 13203.

72. Wang L, Chen J, Leung LT, et al. Use patterns of cigarettes and alternative tobacco products and socioeconomic correlates in Hong Kong secondary school students. Scientific Reports 2021; 11: 17253.

73. Wu YS, Wang MP, Ho SY, et al. Heated tobacco products use in Chinese adults in Hong Kong: a population-based cross-sectional study. Tobacco Control 2020; 29: 277-281.

74. Xia W, Li WHC, Luo YH, et al. The association between heated tobacco product use and cigarette cessation outcomes among youth smokers: A prospective cohort study. J Subst Abuse Treat 2022; 132: 108599.

75. Yi J, Lee CM, Hwang S-S, Cho S-I. Prevalence and predictors of heated tobacco products use among male ever smokers: results from a Korean longitudinal study. BMC Public Health 2021; 21: 316.

76. Zhu S-H, Ong J, Wong S, et al. Early adoption of heated tobacco products resembles that of e-cigarettes. Tobacco Control 2022; 31: e35-e40.
